# Anatomically and Functionally Distinct Lung Mesenchymal Populations Marked by Lgr5 and Lgr6

**DOI:** 10.1016/j.cell.2017.07.028

**Published:** 2017-09-07

**Authors:** Joo-Hyeon Lee, Tuomas Tammela, Matan Hofree, Jinwook Choi, Nemanja Despot Marjanovic, Seungmin Han, David Canner, Katherine Wu, Margherita Paschini, Dong Ha Bhang, Tyler Jacks, Aviv Regev, Carla F. Kim

**Affiliations:** 1 Stem Cell Program and Divisions of Hematology/Oncology and Pulmonary & Respiratory Diseases, Boston Children’s Hospital, Boston, MA 02115, USA; 2 Harvard Stem Cell Institute, Cambridge, MA 02138, USA; 3 Department of Genetics, Harvard Medical School, Boston, MA 02115, USA; 4 Wellcome Trust/Medical Research Council Stem Cell Institute, University of Cambridge, Tennis Court Road, Cambridge CB2 1QR, UK; 5 Department of Physiology, Development and Neuroscience, University of Cambridge, Cambridge CB2 3DY, UK; 6 David H. Koch Institute for Integrative Cancer Research, Massachusetts Institute of Technology, Cambridge, MA 02142, USA; 7 Broad Institute of MIT and Harvard, Cambridge, MA 02142, USA; 8 Wellcome Trust/Cancer Research UK Gurdon Institute, University of Cambridge, Tennis Court Road, Cambridge CB2 1QN, UK; 9 Department of Cancer Biology, Abramson Family Cancer Research Institute, University of Pennsylvania School of Medicine, Philadelphia, PA 19104, USA; 10 Howard Hughes Medical Institute, Department of Biology, Massachusetts Institute of Technology, Cambridge, MA 02139, USA

**Keywords:** mesenchymal cells, bronchiolar epithelium, alveolar epithelium, lung stem cells, lung, differentiation, niche, Wnt signaling

## Abstract

The diversity of mesenchymal cell types in the lung that influence epithelial homeostasis and regeneration is poorly defined. We used genetic lineage tracing, single-cell RNA sequencing, and organoid culture approaches to show that Lgr5 and Lgr6, well-known markers of stem cells in epithelial tissues, are markers of mesenchymal cells in the adult lung. Lgr6^+^ cells comprise a subpopulation of smooth muscle cells surrounding airway epithelia and promote airway differentiation of epithelial progenitors via Wnt-Fgf10 cooperation. Genetic ablation of Lgr6^+^ cells impairs airway injury repair in vivo. Distinct Lgr5^+^ cells are located in alveolar compartments and are sufficient to promote alveolar differentiation of epithelial progenitors through Wnt activation. Modulating Wnt activity altered differentiation outcomes specified by mesenchymal cells. This identification of region- and lineage-specific crosstalk between epithelium and their neighboring mesenchymal partners provides new understanding of how different cell types are maintained in the adult lung.

## Highlights

• Lgr5 and Lgr6 mark mesenchymal cells in adult lungs
• Single-cell transcriptome analysis defines mesenchymal heterogeneity
• Distinct mesenchymal niches drive airway and alveolar differentiation
• Wnt activity affects epithelial differentiation specified by mesenchymal cells

## Graphical Abstract

**Figure:**
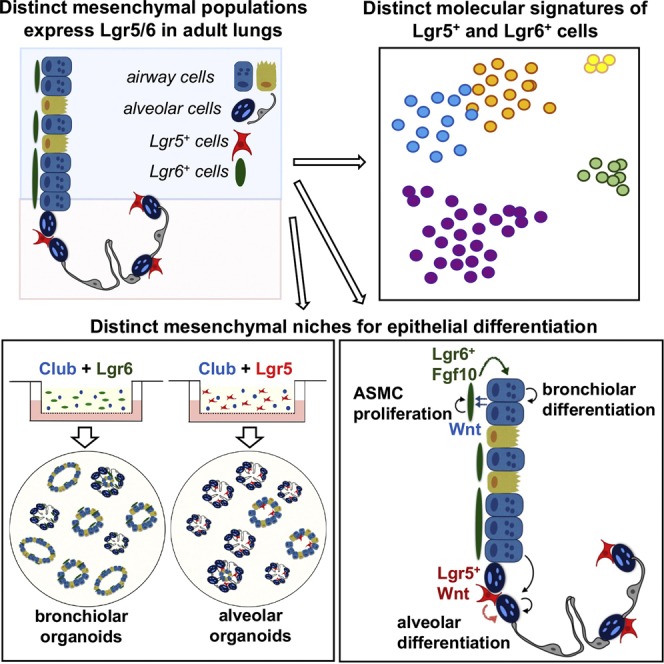

## Teaser

Heterogeneous mesenchymal cell populations in the lung play a central role in epithelial maintenance and alveolar differentiation.

## Introduction

Homeostasis and injury repair of the adult lung epithelium involve the active engagement of epithelial cell populations that reside in distinct anatomical locations. In the distal lung, multiple progenitor populations have been shown to participate in the repair process. The different anatomical locations of diverse epithelial progenitor cells in the lung make it likely that distinct stromal factors regulate the behavior of these cells. However, understanding the precise molecular mechanisms influencing progenitor cells is precluded by limited knowledge of stromal cell identities in the lung.

Defining the identities and behavior of lung mesenchymal cells is challenging due to the lack of defined markers for these populations. During lung development, the mesenchymal progenitors undergo regionally distinct differentiation programs, giving rise to airway and vascular smooth muscle, alveolar fibroblasts, endothelium, and pericytes, among others (McCulley et al., 2015). Clonal analysis illustrated the diversity of mesenchymal progenitors (Kumar et al., 2014). Mesenchyme expressing fibroblast growth factor 10 (Fgf10), glioma-associated oncogene 1 (Gli1), and Axin2 contribute to smooth muscle and alveolar fibroblast-like cells (El Agha et al., 2014, Li et al., 2015, Mailleux et al., 2005, Moiseenko et al., 2017). However, the information on the spatial heterogeneity and behavior of mesenchymal cells that impact epithelial progenitors in lung regeneration and repair remains unclear.

In the airway epithelium of the adult murine distal lungs, club cells (formerly known as Clara cells) function as progenitors that can both self-renew and produce differentiated ciliated cells at steady state (Rawlins et al., 2009). Following airway injury using naphthalene, which abolishes Cyp2f2-expressing club cells, the surviving club cells can divide and regenerate the airway epithelium (Hogan et al., 2014). Lineage-tracing approaches showed that cells expressing the club cell marker CCSP, encoded by the *Scgb1a1* gene, are also capable of giving rise to alveolar lineage cells following bleomycin-induced alveolar damage (Rock et al., 2011). However, little is known about the precise mechanisms regulating club cell behavior during repair and regenerative processes.

Wnt signals function in development and regeneration of the lung (Cardoso and Lü, 2006, Hogan et al., 2014), whereas little Wnt activity is documented in the normal adult lung. Recent studies have uncovered a small family of 7-transmembrane receptors, leucine-rich repeat-containing G protein-coupled receptor-5 (Lgr5) family, comprising Lgr4, Lgr5, and Lgr6 (Clevers et al., 2014). Lgr5 is specifically expressed in epithelial stem cells in multiple tissues, including the intestine, liver, and skin (Barker et al., 2007, Barker et al., 2010, Huch et al., 2013, Jaks et al., 2008). Lgr6 expression has been reported in bipotent skin progenitor cells (Snippert et al., 2010). More recently, Wnt-responsive cells expressing Lgr5 were reported to be highly proliferative and progressive in lung adenocarcinoma (Tammela et al., 2017).

Here, we used single-cell RNA sequencing (scRNA-seq), lineage tracing, and organoid cultures to characterize adult lung mesenchymal populations marked by Lgr5 and Lgr6. Lgr6-expressing cells were found surrounding bronchiolar epithelia and in the alveolar space, whereas Lgr5-expressing cells were largely alveolar. Ex vivo organoid co-culture of Scgb1a1 lineage-labeled cells with Lgr6-expressing cells revealed the Lgr6+ cells direct airway differentiation of Scgb1a1^+^ progenitors. In contrast, Lgr5-expressing mesenchymal cells promote alveolar differentiation via activation of Wnt pathway. These results demonstrate that region-specific crosstalk between airway stem cells and adjacent mesenchymal cells is required to maintain proper tissue integrity.

## Results

### Lgr5 and Lgr6 Mark Distinct Mesenchymal Cell Populations in Adult Lung

**Figure 1:**
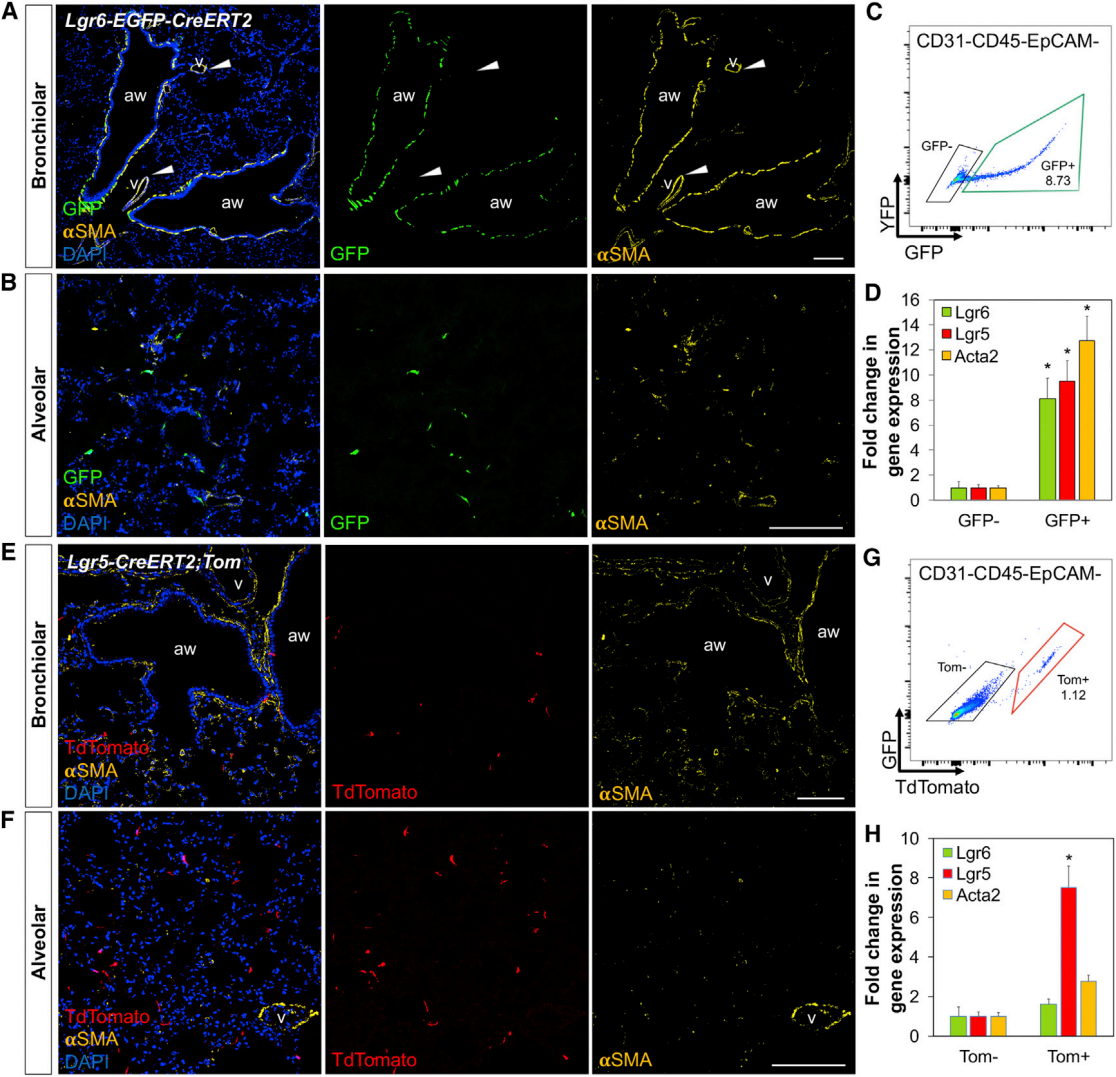
Distinct Mesenchymal Lineages Expressing Lgr5 and Lgr6 in Adult Lungs (A and B) Representative confocal images showing expression patterns of Lgr6 in adult distal lungs: GFP (green); α-SMA (yellow); and DAPI (blue) in lung tissue sections from *Lgr6-EGFP-CreERT2* mice. Arrowheads indicate vascular smooth muscle cells expressing α-SMA^+^. aw, airway; v, blood vessel. (C) Representative profile of FACS-sorted EGFP^+^ populations from *Lgr6-EGFP-CreERT2* mice for qPCR analysis. (D) Validation of differential expression of *Lgr5*, *Lgr6*, and *Acta2* in Lgr6^+^ and Lgr6^−^ cells by qPCR analysis. Expression from Lgr6^+^ cells is shown as fold change relative to Lgr6^−^ cells set to 1, followed by normalization to *Gapdh*. (E and F) Representative confocal images showing expression patterns of Lgr5 in adult distal lungs: Tdtomato (red); α-SMA (yellow); and DAPI (blue) in lung tissue sections from *Lgr5-CreERT2*;*R26-Tom* mice, followed by Tamoxifen injection. aw, airway; v, blood vessel. (G) Representative profile of FACS-sorted TdTomato^+^ populations from *Lgr5-CreERT2*;*R26-Tom* mice for qPCR analysis. Sorting scheme is same as in (C). (H) Validation of differential expression of *Lgr5*, *Lgr6*, and *Acta2* in Lgr5^+^ and Lgr5^−^ cells by qPCR analysis. Normalized as in (D). The scale bars represent 100 μm. Data presented are the mean of three independent experiments with triplicates. Error bars indicate SD (^∗^p < 0.001). See also Figure S1.

To investigate the functional role of Lgr5 and Lgr6 in adult lungs, we characterized Lgr6 expression in the lung using *Lgr6-EGFP-IRES-CreERT2* knockin mice, in which EGFP marks cells with active expression of the *Lgr6* locus (Snippert et al., 2010). Unexpectedly, rather than marking epithelial cells, Lgr6-expressing cells were found throughout the lung mesenchyme surrounding the conducting airways. Immunohistochemistry showed that these cells express α-smooth muscle actin (α-SMA) (encoded by *Acta2*), a marker of smooth muscle cells (Figure 1A). Notably, no Lgr6 expression was observed in vascular smooth muscle cells (VSMCs) (arrowhead, Figure 1A). In the alveolar regions, we found scattered EGFP-positive cells (GFP^+^) that are negative for α-SMA (Figure 1B). Fluorescence-activated cell sorting (FACS) analysis revealed that 9.12% ± 1.42% of resident mesenchymal cells (GFP^+^/CD31^−^CD45^−^EpCAM^−^) express Lgr6 in adult lungs (Figure 1C). qPCR confirmed that these populations robustly express *Lgr6* and *Acta2*. We also detected *Lgr5* expression in the Lgr6^+^ cells, suggesting Lgr6 may mark cell populations expressing Lgr5 (Figure 1D).

**Figure S1:**
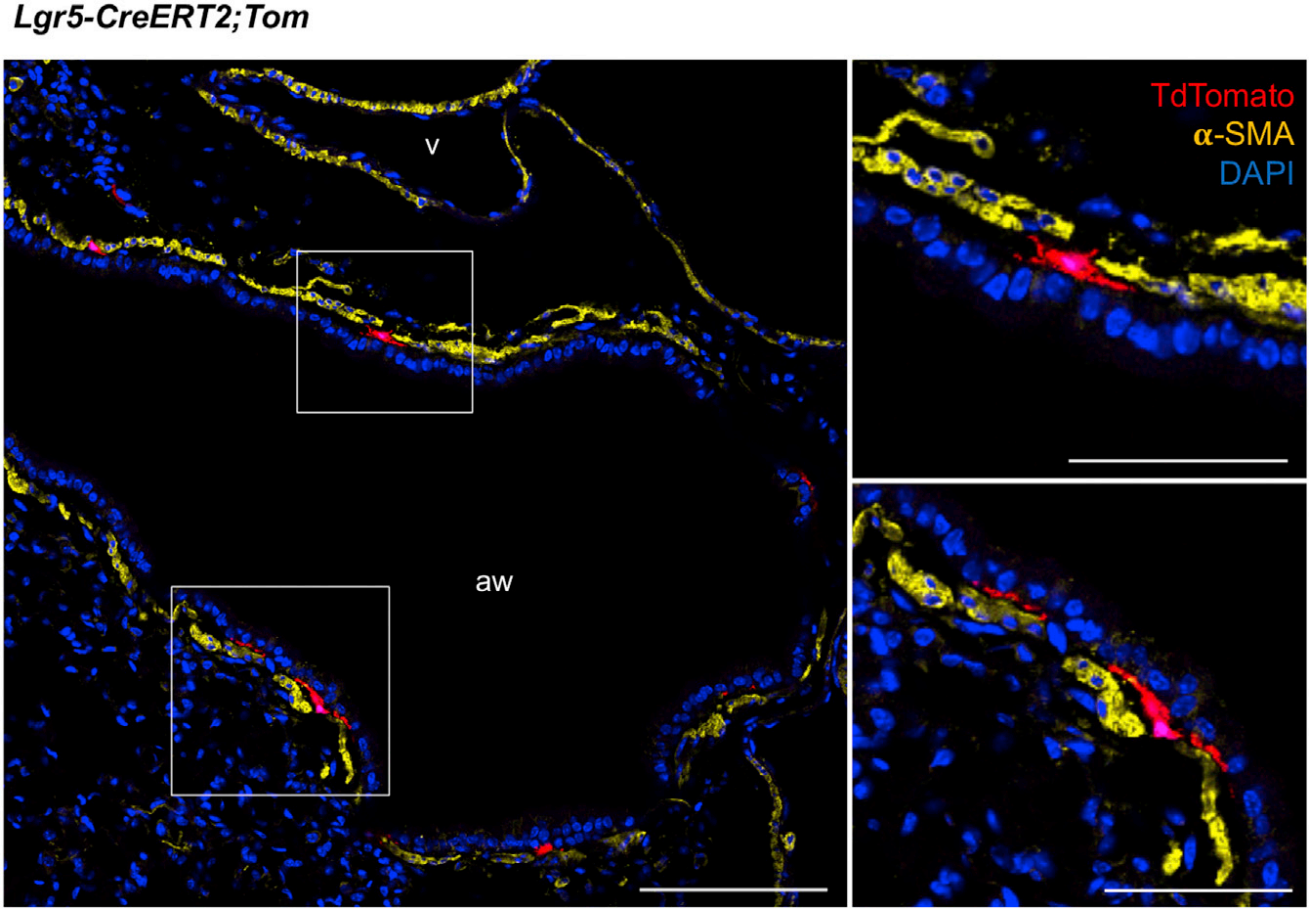
Lgr5 Marks Mesenchymal Lineages around Conducting Airways, Related to Figure 1 Representative confocal images showing expression of Lgr5 in mesenchymal cells around airway epithelium in adult distal lungs: Tdtomato (for Lgr5, red), α-SMA (yellow), and DAPI (blue) in lung tissue sections from *Lgr5-CreERT2;R26-Tom* mice, following by Tamoxifen injection (Tmx, 0.2mg/g x 4). Scale bars, low magnification 200um; high magnification 50um.

We next utilized *Lgr5-IRES-CreERT2* mice that were crossed to a *Rosa26-lox-stop-lox-TdTomato* reporter allele (hereafter, *Lgr5-CreERT2*;*R26-Tom*) to investigate expression of Lgr5 in adult lungs. In contrast with Lgr6^+^ cells, the majority of lineage-labeled Lgr5^+^ cells were located exclusively in the alveolar compartments and none of the lineage-labeled cells were airway smooth muscle cells (ASMCs) (Figures 1E and 1F). A small number of cells that were negative for α-SMA were found near airways (Figure S1). FACS analysis indicated that 1.24% ± 0.42% of resident lung mesenchymal cells (Tom^+^/CD31^−^CD45^−^EpCAM^−^) were lineage-labeled by Lgr5 (Figure 1G). In contrast to the high expression level of Lgr5 in Lgr6^+^ cells, expression of *Lgr6* and *Acta2* was not highly enriched in the cell populations labeled by Lgr5 (Figures 1D and 1H). These results suggest that Lgr5 and Lgr6 mark distinct mesenchymal lineages in adult lungs; the majority of Lgr6^+^ cells are ASMCs, whereas Lgr5^+^ cells are found primarily in the alveolar regions.

### Heterogeneity of Mesenchymal Populations Expressing Lgr5 and Lgr6 in Adult Lungs

**Figure 2:**
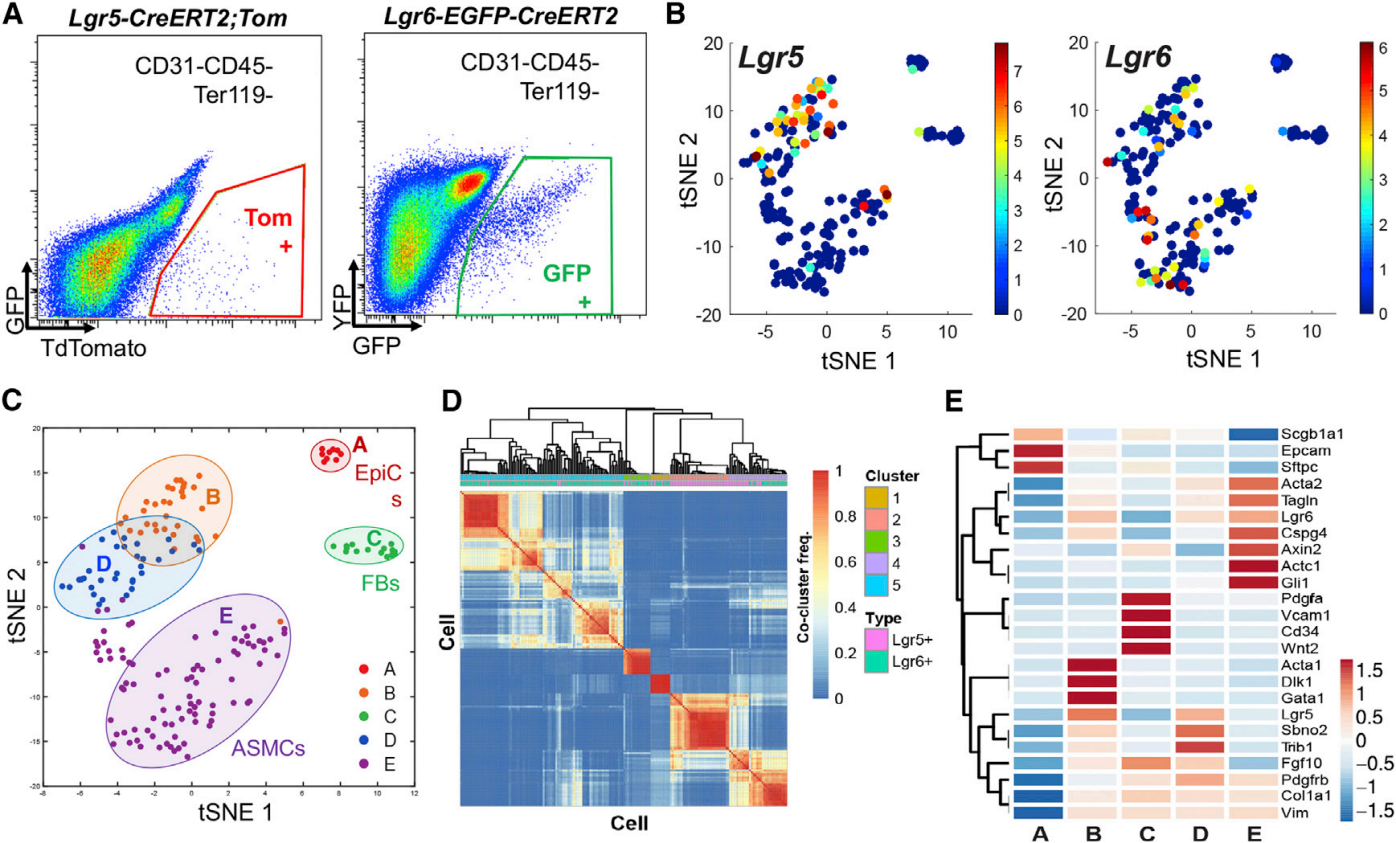
Single-Cell Transcriptome Analysis Distinguishes Various Cell Lineages Labeled by Lgr5 and Lgr6 (A) Representative profile of FACS-sorted GFP^+^ populations for Lgr6-expressing cells from *Lgr6-EGFP-CreERT2* mice (left) and Tom^+^ populations for Lgr5-expressing cells from *Lgr5-CreERT2*;*R26-Tom* mice post-induction (right) for single-cell RNA sequencing. (B and C) T-stochastic neighbor embedding (tSNE) plot of 182 individual cells isolated in (A) (dots), where cells are either colored by the expression of Lgr5 (B, left) or Lgr6 (B, right; color bar, log_2_(TPM+1), left) or by their cluster assignment (C). (D) Consensus clustering. Heatmap shows for each cell (rows and columns) and the frequency of times. Pairs of cells are clustered into the same cluster in 1,000 clustering applied to 1,000 random subsamples (color bar, 0, blue; 1, red). The matrix is hierarchically clustered (dendrogram, top). The final consensus cell clusters are marked by color code (A–E). Lgr5^+^ and Lgr6^+^ cells are marked in pink and green. (E) Heatmap showing the relative average expression for selected marker genes across the 5 clusters. See also Figure S2.

**Figure S2:**
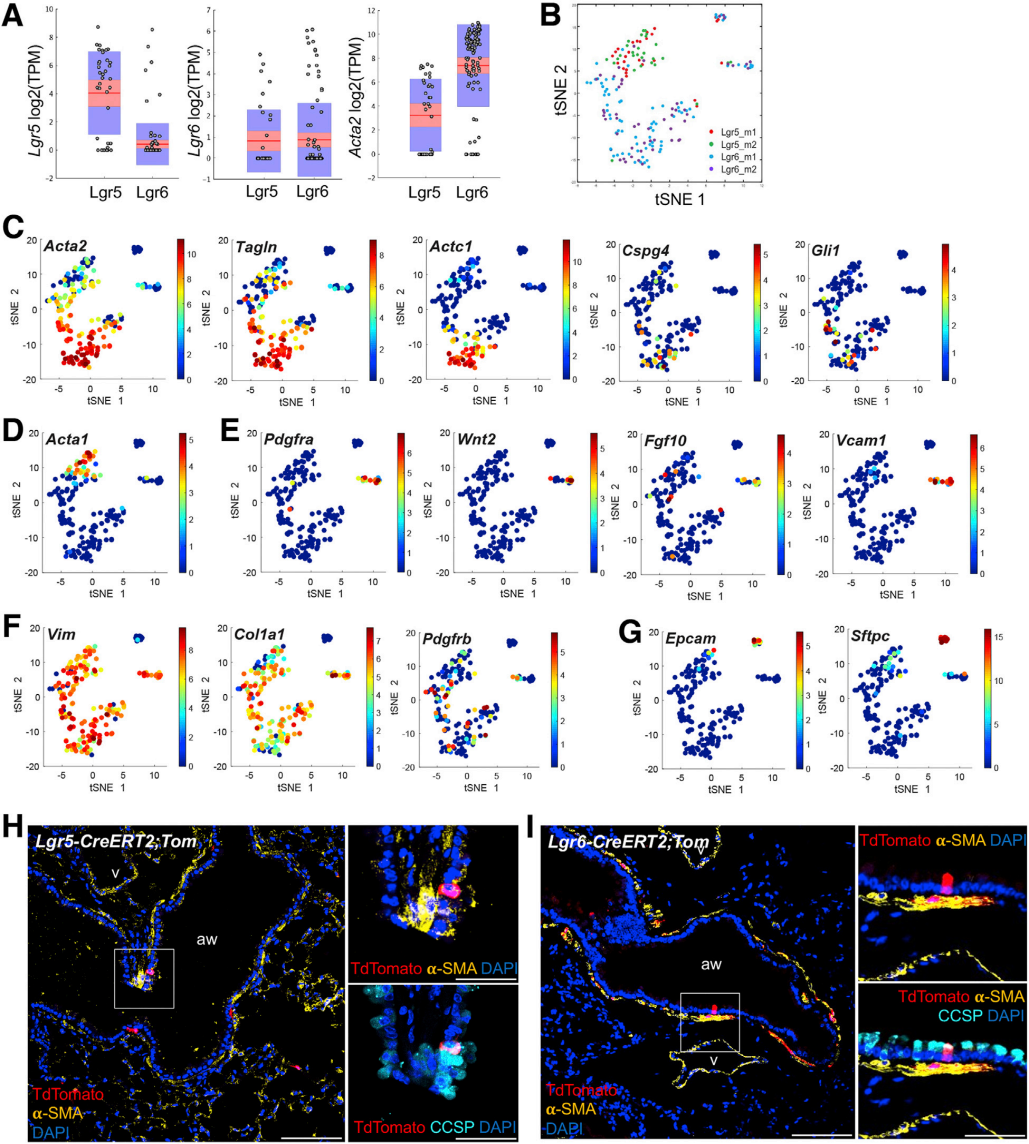
Heterogeneity of Lgr5- and Lgr6-Expressing Cells, Related to Figure 2 (A) Expression of *Lgr5*, *Lgr6*, and *Acta2* in individual cells analyzed by single cell RNA-seq. Shown are boxplots of the distribution of expression levels (log_2_(TPM+1)) for *Lgr5* (left panel), *Lgr6* (middle panel), and *Acta2* (right panel) in Lgr5^+^ (left bar) and Lgr6^+^ (right bar) cells (x axis). (B) T-Stochastic neighbor embedding (tSNE) plot of 182 individual Lgr5^+^ (red and green, two replicates) and Lgr6^+^ (blue and purple, two replicates) cells, after quality filtering. (C–G) tSNE plots as in (B) but where cells are colored by the expression of specific genes, as marked. (H and I) Representative confocal images showing expression of Lgr5 and Lgr6 in airway epithelial cells in adult distal lungs: Tdtomato (for Lgr5 or Lgr6, red), CCSP (cyan), and DAPI (blue) in lung tissue sections from *Lgr5-CreERT2;R26-Tom* (H) and *Lgr6-CreERT2;R26-Tom* (I) mice, following by Tamoxifen injection (Tmx, 0.2mg/g x 4). Scale bars, low magnification 200um; high magnification 50um.

To characterize the mesenchymal lineages labeled by Lgr5 and Lgr6, we performed scRNA-seq of individual cells isolated from *Lgr5-CreERT2*;*R26-Tom* and *Lgr6-EGFP-CreERT2* mice (two replicates of each; Figure S2B). We purified single-cell suspensions of dissociated Lgr5^+^ and Lgr6^+^ cells by FACS sorting with depletion of endothelial and immune cells (Lgr5, CD31^−^CD45^−^CD11b^−^TER119^−^Tom^+^; Lgr6, CD31^−^CD45^−^CD11b^−^TER119^−^GFP^+^; Figure 2A). We analyzed profiles from 182 mesenchymal cells that passed strict quality-control thresholds (STAR Methods) and used a community detection clustering algorithm on *k*-nearest neighbor (k-NN) cell graph, created from random subsamples of the data, to identify robust clusters by consensus clustering (STAR Methods).

We identified five robust clusters of cell populations with distinct transcriptional programs (clusters A–E; Figures 2B–2D and S2B). Using marker genes known to be expressed in various mesenchymal cells, we distinguished Lgr5- and Lgr6-expressing cell types that are regionally distributed. Specifically, cluster E cells had a distinctive high expression of *Lgr6* and *Acta2* but low expression of *Lgr5*, suggesting this is the Lgr6-expressing ASMCs population (Figures 2B–2E, S2B, and S2C). Cells in the cluster also highly expressed various mesenchymal genes, such as *Cspg4*, *Tagln*, and *Gli1*. Clusters B and D cells exhibited enriched expression of Lgr5. Some of the cells in these two clusters showed considerable Lgr6 expression, suggesting that this population contains alveolar mesenchymal cells labeled by Lgr5 and Lgr6 (Figures 2B–2E, S2B, and S2D). A distinct small population, cluster C, highly expresses genes associated with alveolar fibroblasts: *Pdgfra*; *Wnt2*; *Fgf10*; and *Vcam1* (Figures 2B–2E, S2B, and S2E). Cells in all clusters expressing Lgr5 or Lgr6 also expressed general mesenchymal markers, such as *Col1a1*, *Vimentin*, and *Pdgfrb* (Figure S2F). Cluster A, a distinct small cluster of cells, expressed the epithelial marker EpCAM and lung epithelial lineage markers, such as *Scgb1a1* (club cell marker) and *Sftpc* (alveolar type II cell marker; Figures 2B–2E, S2B, and S2G). Lineage-tracing studies confirmed that there are rare cells expressing CCSP in lineage-labeled Lgr5^+^ and Lgr6^+^ cells (Figures S2H and S2I). Each of the cell populations suggested by cluster analysis expressed a unique gene expression signature (Figures 2D and 2E). Taken together, scRNA-seq analysis shows that the cellular heterogeneity of lung mesenchymal cells expressing Lgr5 and Lgr6 is associated with distinct and separable transcriptional programs.

### Long-Term Tracing of Lgr6^+^ Mesenchymal Cells in Adult Lungs in Homeostasis

**Figure 3:**
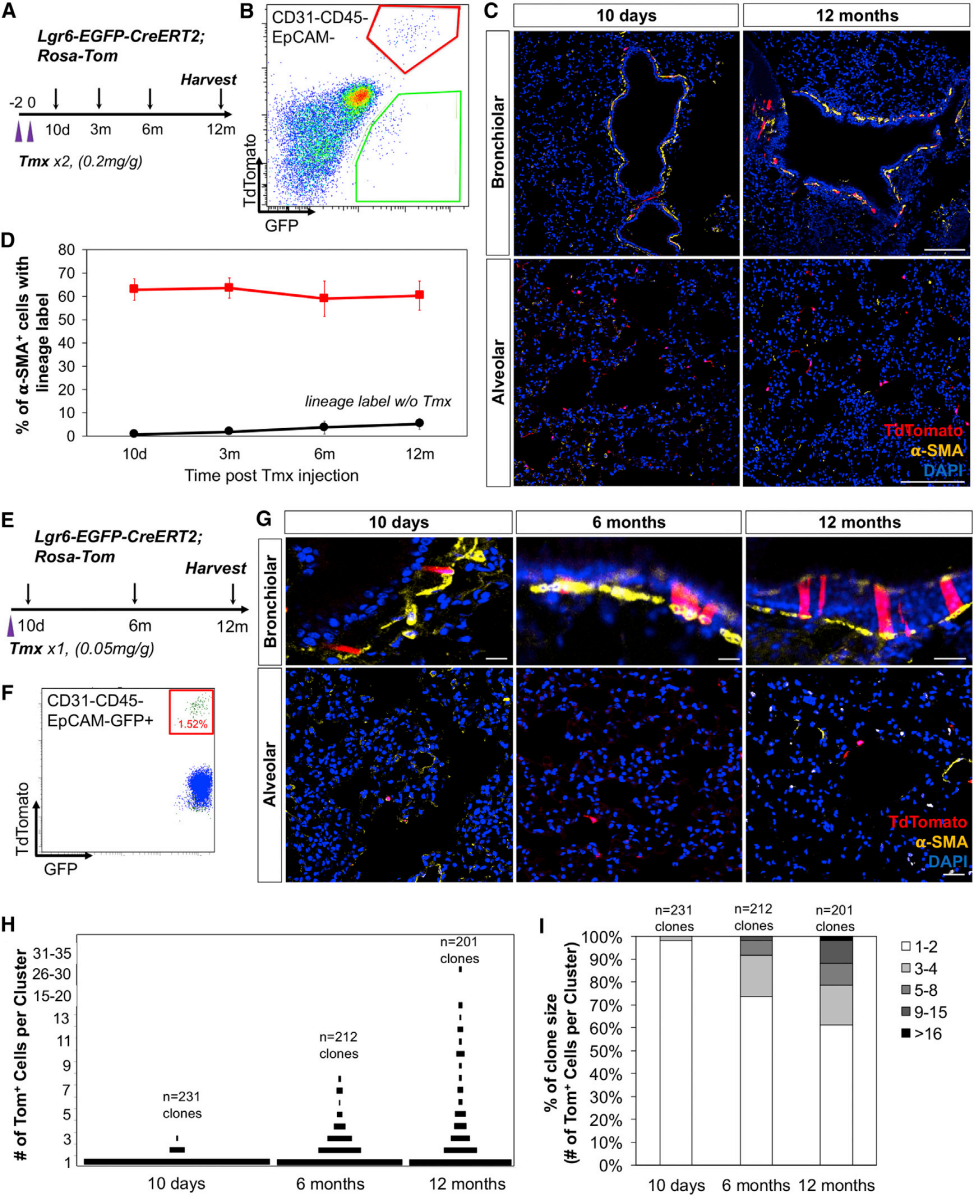
Proliferative Potential of Lineage-Labeled Lgr6+ Cells in Homeostatic Lungs (A and E) Schematics of the *Lgr6-EGFP-CreERT2*;*R26-Tom* lineage-tracing experiment. (B and F) Representative flow cytometry analysis showing the recombination efficiency of lineage-labeled Lgr6^+^ cells after Tmx induction. (C) Representative confocal images showing lineage-labeled Lgr6^+^ cells in adult lungs at 10 days and 12 months after Tmx induction: Tdtomato (red); α-SMA (yellow); and DAPI (blue). (D) Graphs to show the mean percentage of lineage-labeled Lgr6^+^ airway smooth muscle cells (ASMCs) following Tmx exposure (red circles). Control animals receive vehicle alone, and low recombination is seen without Tmx injection (black circles). (G) Representative confocal images showing proliferative expansions of lineage-labeled Lgr6^+^ cells in lung homeostasis: Tdtomato (red); α-SMA (yellow); and DAPI (blue). (H and I) Size distribution of clusters at indicated time points post-induction. Length of bar represents frequency in (H). n, number of clones scored. The scale bars represent 100 μm (C), 10 μm (G, top), and 50 μm (G, bottom).

To evaluate the cellular behavior of Lgr6^+^ cells in adult lungs, we established *Lgr6-EGFP-CreERT2*;*R26-Tom* mice. To determine whether lineage-labeled Lgr6^+^ cells contribute to ASMC maintenance, we measured the proportion of lineage-labeled Lgr6^+^ cells that are positive for α-SMA cells over time (Figure 3A). FACS analysis showed that recombination occurred in 62.7% ± 4.3% of Lgr6-expressing cells (Tom^+^GFP^+^/GFP^+^) at 10 days after final Tmx injection (Figure 3B). As shown in Figures 3C and 3D, the proportion of lineage-labeled Lgr6^+^α-SMA^+^ ASMCs remained constant over the 12-month chase period. To further explore whether Lgr6^+^ cells are resting cells or undergo proliferation in the steady state, *Lgr6-EGFP-CreERT2*;*R26-Tom* mice were injected with a single low dose of Tmx, which labels only a small proportion of Lgr6^+^ cells (Figure 3F). Lungs were harvested over 12-month chase period, and sections were analyzed with confocal microscopy for the presence of lineage-labeled cells or clusters that span both peri-airway and alveolar compartments (Figure 3E). Single-labeled peri-airway or alveolar cells were widely distributed at 10 days after Tmx administration (Figure 3G). Notably, after approximately 6 months, a few small clusters of lineage-labeled peri-airway cells were observed. Longer chases, up to 12 months, confirmed that lineage-labeled cells are long lived and can be found in clusters, suggesting that there is a subset of Lgr6^+^ cells that are capable of proliferation with a slow rate at steady state. The size distribution of clusters became increasingly heterogeneous, but mean clone size and number of cells composed of clusters increased over time, indicating the proliferative potential of lineage-labeled Lgr6^+^ cells (Figures 3H and 3I). At 10 days post-Tmx induction, 92.02% of clones consisted of single-labeled cells (n = 231 clones, 3 mice; Figures 3G–3I). By 6 months, 38.8% of clones were composed of more than one cell, whereas the rest of clones still consisted of single-labeled cells (2 cells, 20.84%; 3 cells, 11.67%; 4 cells, 5.34%; 5–8 cells, 6.22%; n = 212 clones, 3 mice). At 12 months, 51.12% of clones consisted of more than one cell and 21.03% had five or more cells (up to 30 cells; n = 201 clones, 3 mice). Comparison of cell number in each clone indicated that proliferation rates vary among lineage-labeled cells (Figures 3H and 3I). No lineage-labeled clusters were observed in alveolar compartments even after 12 months at steady state (Figure 3G). These data support the hypothesis that Lgr6^+^ cells contain ASMCs, maintaining the peri-bronchiolar microenvironment in normal lung homeostasis. In addition, Lgr6^+^ peri-bronchiolar cells expand over time, yet the alveolar Lgr6^+^ cells appear to be a quiescent cell population in normal tissue homeostasis.

### Cellular Dynamics in Response to Ablation of Lgr6^+^ Cells

**Figure 4:**
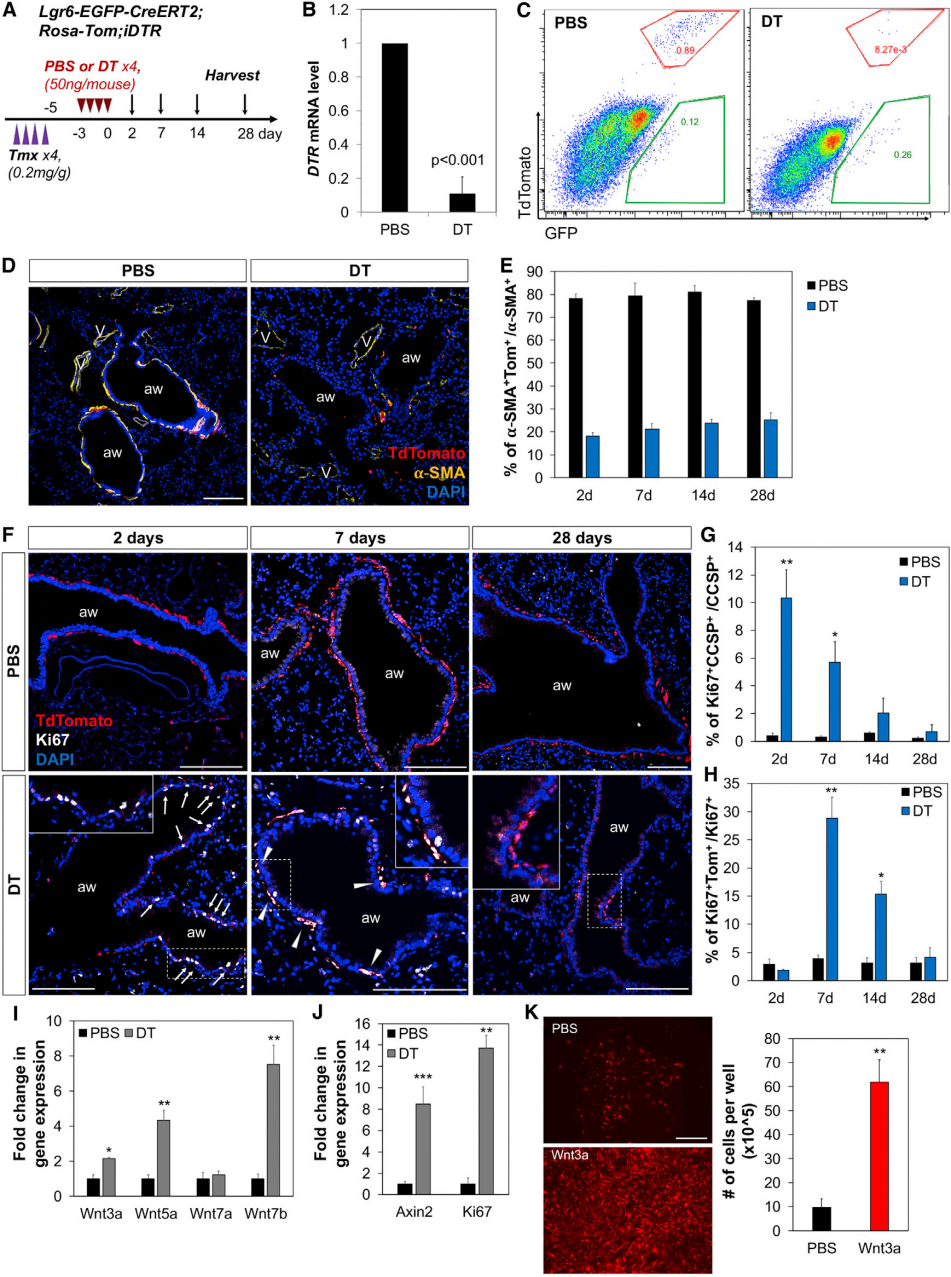
Paired Proliferative Expansion of Lgr6+ Cells and Airway Epithelial Cells after Genetic Ablation of Lgr6+ Cells (A) Schematics of the *Lgr6-EGFP-CreERT2;R26-Tom* lineage-tracing experiment. (B) qPCR analysis for expression of diphtheria toxin receptor (*DTR*) in whole lungs from PBS- and DT-treated mice. Shown is normalized to *Gapdh*. (C) Representative flow cytometry analysis showing the efficient ablation of Lgr6^+^ cells at 2 days after PBS or DT administration. Cells are gated out from CD31^−^CD45^−^EpCAM^−^ populations. (D) Representative confocal images showing the specific ablation of ASMCs marked by Lgr6 at 2 days post-PBS or DT administration: Tdtomato (red); α-SMA (yellow); and DAPI (blue). (E) Graphs to show the mean percentage of lineage-labeled Lgr6^+^ cells that express α-SMA at indicated time points after PBS (black bars) or DT (blue bars) treatment. (F) Representative confocal images showing proliferation of airway epithelial cells and lineage-labeled Lgr6^+^ cells after PBS or DT administration: Tdtomato (red); Ki67 (white); and DAPI (blue). Arrow, Ki67^+^ airway epithelial cells; arrowhead, Ki67^+^Lgr6^+^ cells. (G) Graphs to show the mean percentage of CCSP^+^Ki67^+^ cells that express CCSP at indicated time points after PBS (black bars) or DT (blue bars) treatment. (H) Graphs to show the mean percentage of lineage-labeled Lgr6^+^ cells that express Ki67 at indicated time points after PBS (black bars) or DT (blue bars) treatment. (I and J) qPCR analysis for expression of Wnt ligands in isolated secretory cells (I) and *Axin2* and *Ki67* in isolated lineage-labeled Lgr6^+^ cells (J) from PBS-treated (black bar) and DT-treated (gray bar) lungs. Shown is normalized to *Gapdh*. (K) Representative images of in vitro culture of lineage-labeled Lgr6^+^ cells with PBS or Wnt3a and quantification of Lgr6^+^ cell numbers at five days in culture. Data presented are the mean of experiments from three individual mice per group (A, E, and G–J) or the mean of three independent experiments (K). Error bars indicate SD (^∗^p < 0.01; ^∗∗^p < 0.005; ^∗∗∗^p < 0.001). The scale bars represent 100 μm. See also Figure S3.

**Figure S3:**
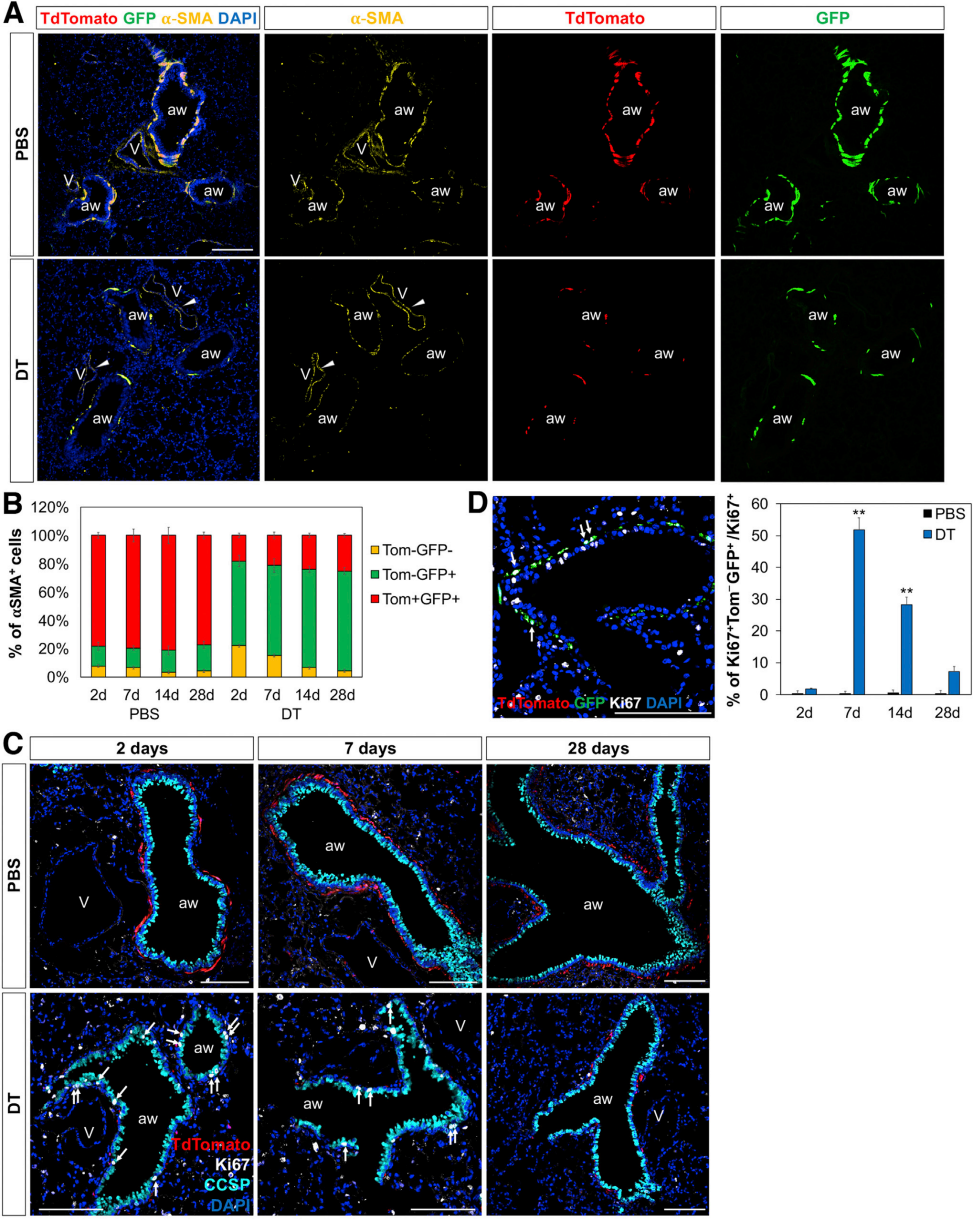
Proliferation of Lgr6-Expressing Cells following Targeted Injury of Lgr6^+^ Cells, Related to Figure 4 (A) Representative confocal images showing the specific ablation of airway smooth muscle cells (ASMCs) marked by Lgr6 at 2 days post PBS (upper panels) or DT (lower panels) administration: Tdtomato (red), α-SMA (yellow), GFP (green), and DAPI (blue). Arrowhead, vascular smooth muscle cells. (B) Graphs to show the mean percentage of lineage-labeled Lgr6^+^ cells (Tom^+^GFP^+^, red bar), non-labeled Lgr6-expressing cells (Tom^–^GFP^+^, green bar), and Lgr6-negative cells (Tom^–^GFP^–^, yellow bar) that express α-SMA at indicated time points after PBS or DT treatment. (C) Representative confocal images showing proliferation of club cells after PBS (upper panels) or DT (lower panels) administration in *Lgr6-CreERT2;R26-Tom;R26-iDTR* mice: Tdtomato (for Lgr6, red), Ki67 (white), CCSP (cyan) and DAPI (blue). Arrow, Ki67^+^ club cells. (D) Representative confocal images (left) and graph (right) showing proliferation of Lgr6-expressing cells (Tom^–^GFP^+^) that were not tagged at 7days after DT administration (left): Tdtomato (red), Ki67 (white), GFP (green), and DAPI (blue). Arrow, Ki67^+^ Lgr6-expressing cells. Graphs to show the mean percentage of non-labeled Lgr6-expressing cells that express Ki67 at indicated time points after PBS (black bars) or DT (blue bars) treatment. Data represent the mean of percentage from three individual mice per group (at least five sections). Error bars indicate standard deviation (^∗∗^p < 0.005). Scale bars, 100um.

We next used a cell-specific injury model compatible with high-resolution imaging to deplete Lgr6^+^ cells in vivo. We crossed mice with an inducible human diphtheria toxin receptor allele (iDTR) to *Lgr6-EGFP-CreERT2*;*R26-Tom* mice; in this system, tamoxifen administration induces expression of DTR, allowing subsequent ablation of Lgr6^+^ cells by DT treatment (Figure 4A). Two days after final DT injection, successful ablation of Lgr6^+^ cells was verified by qPCR on whole lungs for DTR mRNA (Figure 4B). In addition, we detected a substantial reduction in lineage-labeled Lgr6^+^ cells after DT administration compared to vehicle by FACS and immunofluorescence (IF) analysis. Whereas 83.1% ± 3.78% of lineage-labeled Lgr6^+^ cells (Tom^+^GFP^+^/GFP^+^) were observed in control animals, only 13.1% ± 0.42% of lineage-labeled cells were detected in the lungs after DT administration, suggesting efficient ablation of Lgr6^+^ cells (Figure 4C). DT injection decreased α-SMA^+^ ASMCs yet had no appreciable effect on α-SMA^+^ VSMCs, indicating that DT caused the specific ablation of Lgr6-labeled cells and not other cell types (Figures 4D and S3A). Despite the extent of Lgr6^+^ ASMC death, the overall histology of the lung remained remarkably intact at 2, 7, 14, and 28 days post-DT treatment; there was little inflammation and no evidence of airway disruption (Figures 4D, 4F, S3A, and S3C).

We next asked whether Lgr6^+^ cells are capable of replacing ablated ASMCs by tracking surviving lineage-labeled Lgr6^+^ cells after depletion. As expected from our earlier long-term lineage-tracing studies, the proportion of lineage-labeled cells remained relatively constant over the chase period in control lungs (Figures 4E and S3B). In the DT-treated group, the initial proportion of lineage-labeled ASMCs was lower, with only 17.46% ± 3.61% of the α-SMA^+^ ASMCs being labeled at 2 days. This value was slightly increased during repair (Figures 4E and S3B). Notably, all of lineage-labeled Lgr6^+^ cells were GFP positive, indicating the expression of Lgr6 in these cells. A higher proliferation rate of airway epithelial cells was observed in the lungs treated with DT at 2 days post-injection compared with PBS control (Figures 4F, 4G, and S3C). In contrast, a remarkable reduction in lineage-labeled Lgr6^+^ cells was observed at this time point. At 7 days after DT treatment, fewer airway epithelial cells were proliferative, and there was a significant increase in lineage-labeled Lgr6^+^ cells that co-expressed Ki67 (Figures 4F–4H). Of note, Lgr6-expressing cells that were not tagged (Tom^−^GFP^+^) are also capable of expansion, whereas none of Tom^−^GFP^−^α-SMA^+^ ASMCs expressed Ki67 (Figure S3D). The proportion of non-lineage-labeled Lgr6^+^α-SMA^+^ ASMCs (Tom^−^GFP^+^) was also slightly increased over time, whereas that of Lgr6^−^α-SMA^+^ ASMCs (Tom^−^GFP^−^) was reduced during injury repair (Figure S3B). These data indicate that Lgr6^+^ ASMCs are more efficient at injury repair compared to Lgr6^−^ ASMCs. Minimal proliferation was observed in these cells at 28 days after cell depletion, suggesting repair being complete around this time. Notably, lineage-labeled small clusters were seen, indicating the expansion in lineage-labeled Lgr6^+^ASMCs over time (Figure 4F). Together, these results indicate that Lgr6-expressing cells are capable of proliferative expansion after genetic depletion. We next asked whether this expansion would be affected by adjacent airway epithelial cells following targeted injury. Airway secretory cells, including club cells (CD31^−^CD45^−^EpCAM^+^SSEA-1^+^), were isolated from the PBS- and DT-treated lungs at 7 days after ablation of Lgr6^+^ cells. qPCR analysis revealed the higher expression levels of Wnt ligands, such as *Wnt3a*, *Wnt5a*, and *Wnt7b*, in cells from DT-treated mice than in cells from PBS-treated mice (Figure 4I). Of note, isolated Lgr6^+^ cells from the same mice showed a significant increase in *Axin2* and *Ki67* expression post-injury compared to the control cells, suggesting induction of Wnt activation in proliferating Lgr6^+^ ASMCs following targeted injury (Figure 4J). We next assessed whether Wnt signaling induces proliferation of Lgr6^+^ cells in vitro. Isolated lineage-labeled Lgr6^+^ cells were cultured with PBS or Wnt3a. Notably, we observed the remarkable increase of proliferation in lineage-labeled ASMCs, with Wnt3a indicating again that Wnt ligands induce expansion of Lgr6^+^ cells (Figure 4K). Together, these results strongly suggest that Lgr6-expressing cells are capable of proliferation, at least partially stimulated by Wnt ligands from proliferative airway epithelial cells.

### Multi-lineage Organoid Formation of Club Cells with Lgr6^+^ Cells

**Figure 5:**
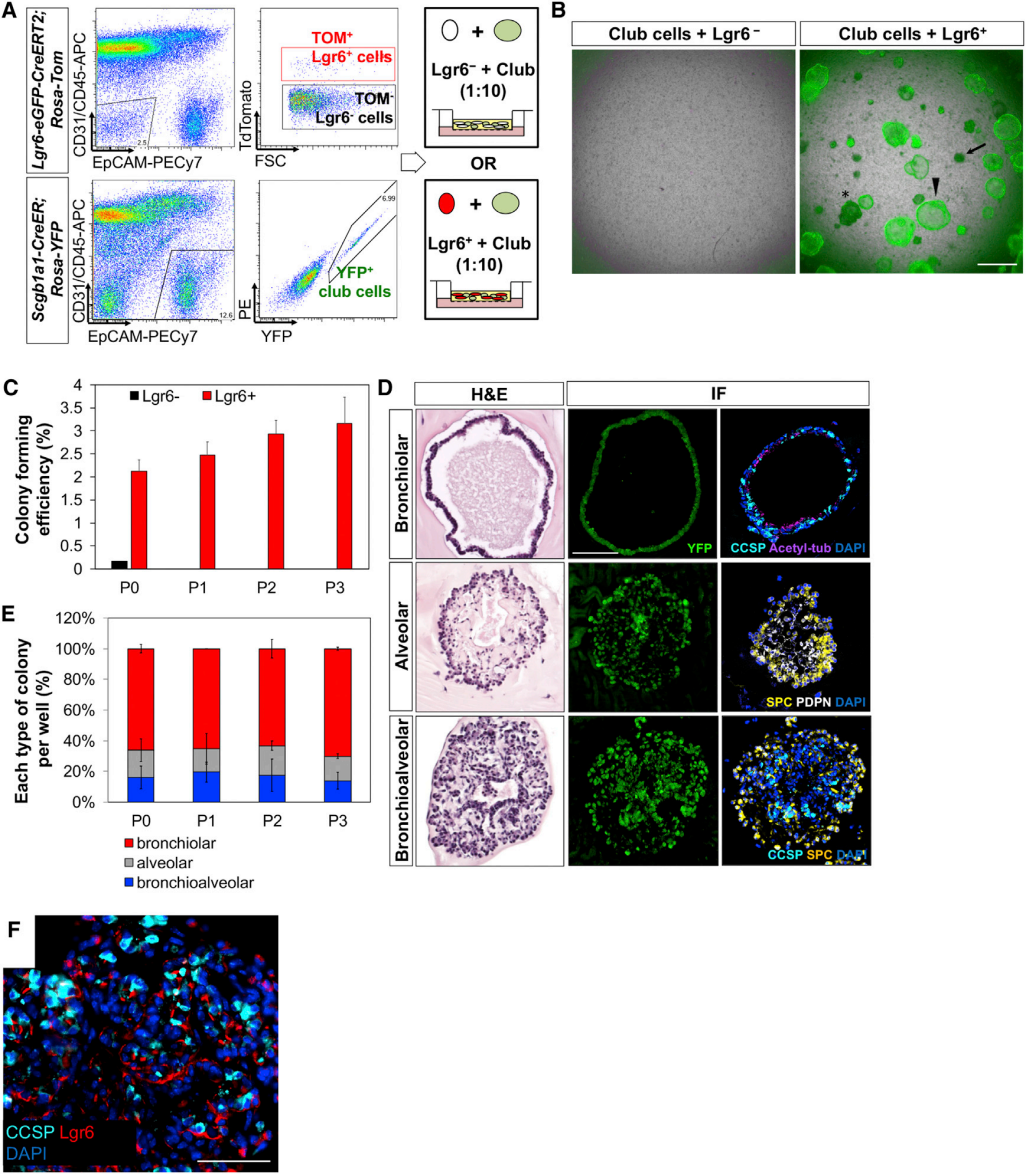
Multi-lineage Differentiation of Lineage-Labeled Scgb1a1^+^ Cells in Organoid Co-culture with Lgr6^+^ Cells (A) Diagram of experimental setup for organoid co-culture of Scgb1a1^+^ cells with Lgr6^−^ and Lgr6^+^ cells. (B) Representative merged images of lung organoids derived from lineage-labeled YFP^+^Scgb1a1^+^ cells co-cultured with Lgr6^−^ or Lgr6^+^ cells in 14-day culture. Note heterotypic colony formation from Scgb1a1^+^ cells. Arrowhead, bronchiolar colony; arrow, alveolar colony; asterisk, bronchioalveolar colony. (C) Colony forming efficiency of Scgb1a1^+^/Lgr6^+^ organoids with serial passages. P0, passage 0; P1, passage 1; P2, passage 2; P3, passage 3. (D) Representative images of organoids from Scgb1a1^+^/Lgr6^+^ co-cultures; bronchiolar (top), alveolar (middle), and bronchioalveolar (bottom) colonies. H&E (left) and IF (right) for YFP (green), CCSP (cyan), acetylated tubulin (purple), SPC (yellow), PDPN (white), and DAPI (blue) are shown. (E) Quantification of each distinct type of colony from Scgb1a1^+^/Lgr6^+^ co-cultures. (F) Representative IF image showing integration of Lgr6^+^ cells within epithelial organoids in (B): CCSP (cyan); TdTomato (for Lgr6, red); and DAPI (blue). Data presented are the mean of three independent experiments with triplicate wells. Error bars indicate SD (C and E). The scale bars represent 500 μm (A) and 100 μm (D and F). See also Figure S4.

**Figure S4:**
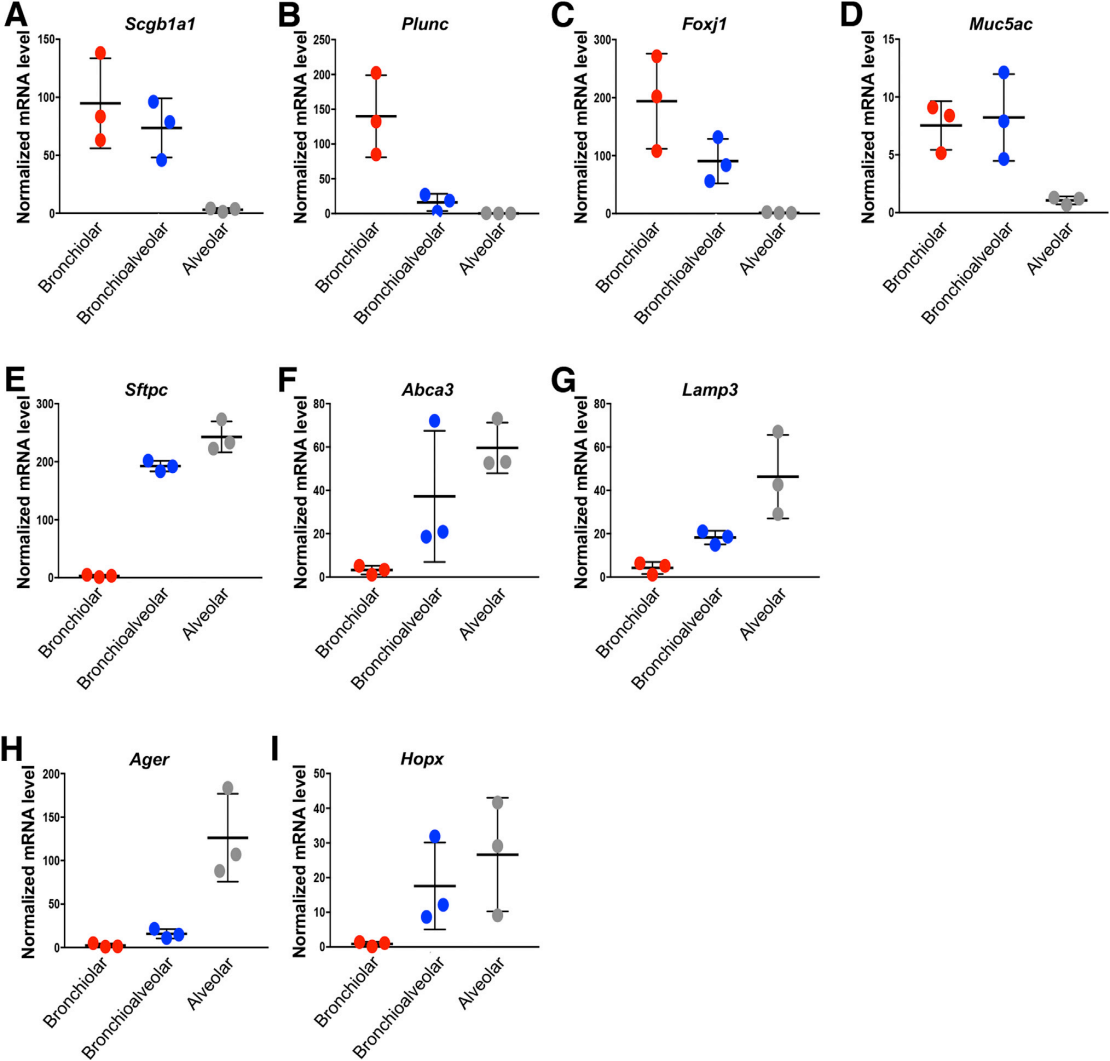
Lgr6^+^ Cells Support Multi-lineage Differentiation of Scgb1a1^+^ Cells, Related to Figure 5 qPCR analysis for expression levels of airway lineage markers (A-D) and alveolar lineage markers (E-I) on individual organoids of different typologies that were derived from lineage-labeled Scgb1a1^+^ cells co-cultured with Lgr6^+^ cells: *Scgb1a1*, *Plunc* for club cells; *Foxj1* for ciliated cells; *Muc5ac* for goblet cells; *Sftpc*, *Abca3*, *Lamp3* for AT2 cells; *Ager*, *Hopx* for AT1 cells. Each graph shows the expression levels of each gene per typology of organoid (n = 3 organoid per typology). Normalized to *Gapdh*.

To functionally interrogate the interactions between Lgr6^+^ mesenchymal cells and lung epithelial cells, we utilized a 3D organoid co-culture system (Lee et al., 2014). Epithelial cells from *Scgb1a1-CreER*; *R26-YFP* animals were isolated by FACS (CD31^−^CD45^−^EpCAM^+^YFP^+^) and co-cultured either with lineage-labeled Lgr6^+^ cells (CD31^−^CD45^−^EpCAM^−^Tom^+^) or with Lgr6^−^ cells (CD31^−^CD45^−^EpCAM^−^Tom^−^) isolated from *Lgr6-EGFP-CreERT2*;*R26-Tom* lungs (Figure 5A). After 14 days in culture, epithelial organoids were observed only in co-cultures that contained lineage-labeled Lgr6^+^ cells; Lgr6^−^ cells did not support organoid formation or cell growth (Figure 5B). Colony-forming efficiency (CFE) at day 14 in culture was 2.12% ± 0.25% in primary culture (Figure 5C). Importantly, Lgr6^+^ cells supported the differentiation of Scgb1a1 lineage-labeled cells into secretory, ciliated cells and alveolar lineage cells, as expected from our in vivo lineage-tracing studies (Figures 5D, 5E, and S4). Morphological, H&E, and IF analysis revealed that three distinct colony types arose in Scgb1a1^+^/Lgr6^+^ co-cultures (Figures 5B, 5D, 5E, and S4), as expected for the FACS-sorted Scgb1a1 lineage-labeled cells based on our previous organoid culture studies (Lee et al., 2014). Large and rounded colonies had a single lumen with secretory and ciliated cells expressing CCSP and acetylated tubulin, respectively (arrowhead, Figures 5B, 5D, and 5E). qPCR analysis in individual organoids also showed higher expression levels of airway lineage markers, such as *Scgb1a1*, *Plunc*, *Foxj1* (ciliated cell marker), and *Muc5ac* (goblet cell marker) in bronchiolar organoids than in alveolar organoids (Figures S4A–S4D). Small and dense colonies showed branching structures composed of surfact protein C (SPC)-expressing AT2 cells in the outer layer and podoplanin (PDPN)-expressing AT1 cells in the inner layer of colonies (arrow, Figures 5B, 5D, and 5E). qPCR analysis on individual alveolar organoids confirmed higher expression levels of alveolar lineage markers, such as *Sftpc*, *Abca3*, *Lamp3* (AT2 cell markers), *Ager*, and *Hopx* (AT1 cell markers) compared to on bronchiolar organoids (Figures S4E–S4I). Mixed colonies contained columnar epithelial cells expressing CCSP in the interior and cells expressing SPC in the peripheral layer (asterisk, Figures 5B, 5D, and 5E). To determine whether Scgb1a1 lineage-labeled cells continue to self-renew and differentiate with Lgr6^+^ cells in culture, day 14 cultures were dissociated and YFP^+^ cells were replated with freshly isolated lineage-labeled Lgr6^+^ cells. CFE was 2.47% ± 0.3% after the first passage and 2.93% ± 0.29% after the second passage (p = n.s. for P1 versus P2; Figure 5C). Moreover, colonies showed the same morphological and histological characteristics over passages up to passage 3 (Figure 5E), indicating differentiation potential of Scgb1a1^+^ cells are maintained in co-culture with lineage-labeled Lgr6^+^ cells. Moreover, IF confirmed the presence of lineage-labeled Lgr6^+^ cells within and around the colonies in close proximity to airway epithelial cells (Figure 5F). Together, these data identify mesenchymal Lgr6^+^ cells as a stromal cell subtype that provide the niche for club cells to maintain their stem cell capacity, including self-renewal and multi-lineage differentiation.

### Crosstalk between Lgr6^+^ Cells and Epithelial Cells in Airway Regeneration In Vivo

**Figure 6:**
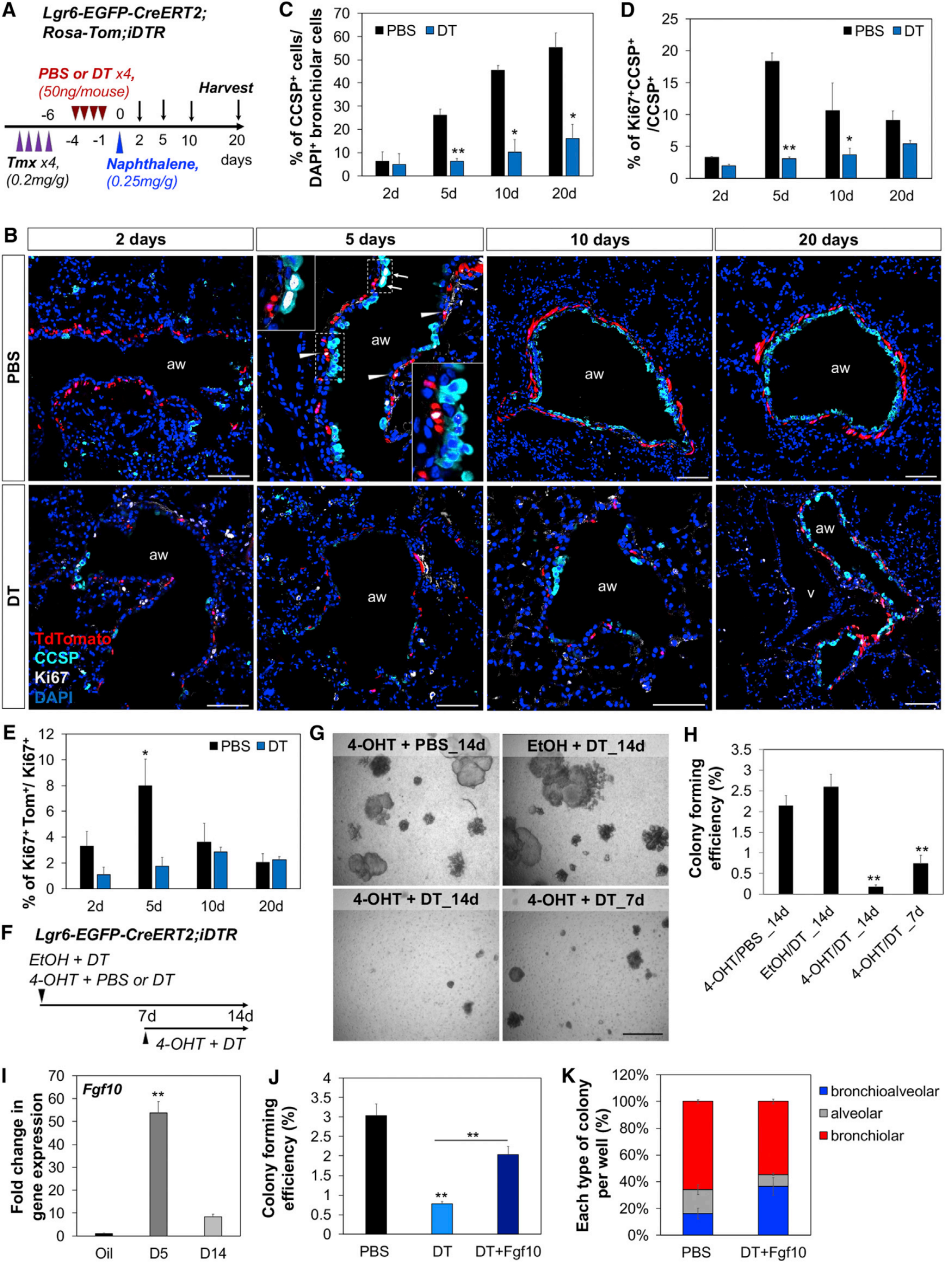
Impaired Airway Regeneration after Ablating Lgr6^+^ Cells (A) Schematics of the *Lgr6-EGFP-CreERT2*;*R26-Tom* lineage-tracing experiment after airway injury. (B) Representative confocal images showing extent of club cell injury or repair with (PBS, top) or without Lgr6^+^ cells (DT, bottom) after naphthalene administration by IF analysis: Tdtomato (for Lgr6, red); Ki67 (white); CCSP (cyan); and DAPI (blue). Arrow, Ki67^+^ airway epithelial cells; arrowhead, Ki67^+^Lgr6^+^ cells. (C–E) Quantification of naphthalene injury repair and expansion of Lgr6^+^ cells in lungs with or without Lgr6^+^ cells. (C) For club cell analysis, the percentage of DAPI-positive bronchiolar cells also positive for CCSP is assessed at indicated time points. (D and E) The mean percentage of club cells or lineage-labeled Lgr6^+^ cells that co-express Ki67 is assessed at indicated time points. (F) Experimental scheme of depleting Lgr6-expressing cells in co-culture of Scgb1a1^+^ cells. (G and H) Representative phase-contrast images (G) and colony forming efficiency (H) of primary lung organoids in (F). (I) qPCR analysis for the expression of *Fgf10* in isolated lineage-labeled Lgr6^+^ cells from naphthalene-treated lungs. Shown is normalized to *Gapdh*. (J and K) Colony forming efficiency (J) and quantification of each distinct colony type (K) of lung organoids with addition of DT and Fgf10 at day 7 in cultures. Data presented are the mean of experiments from three individual mice per group (D, E, and I) or the mean of three independent experiments with triplicate wells (H, J, and K). Error bars indicate SD (^∗^p < 0.01; ^∗∗^p < 0.005). The scale bars represent 50 μm (B) and 500 μm (G). See also Figure S5.

**Figure S5:**
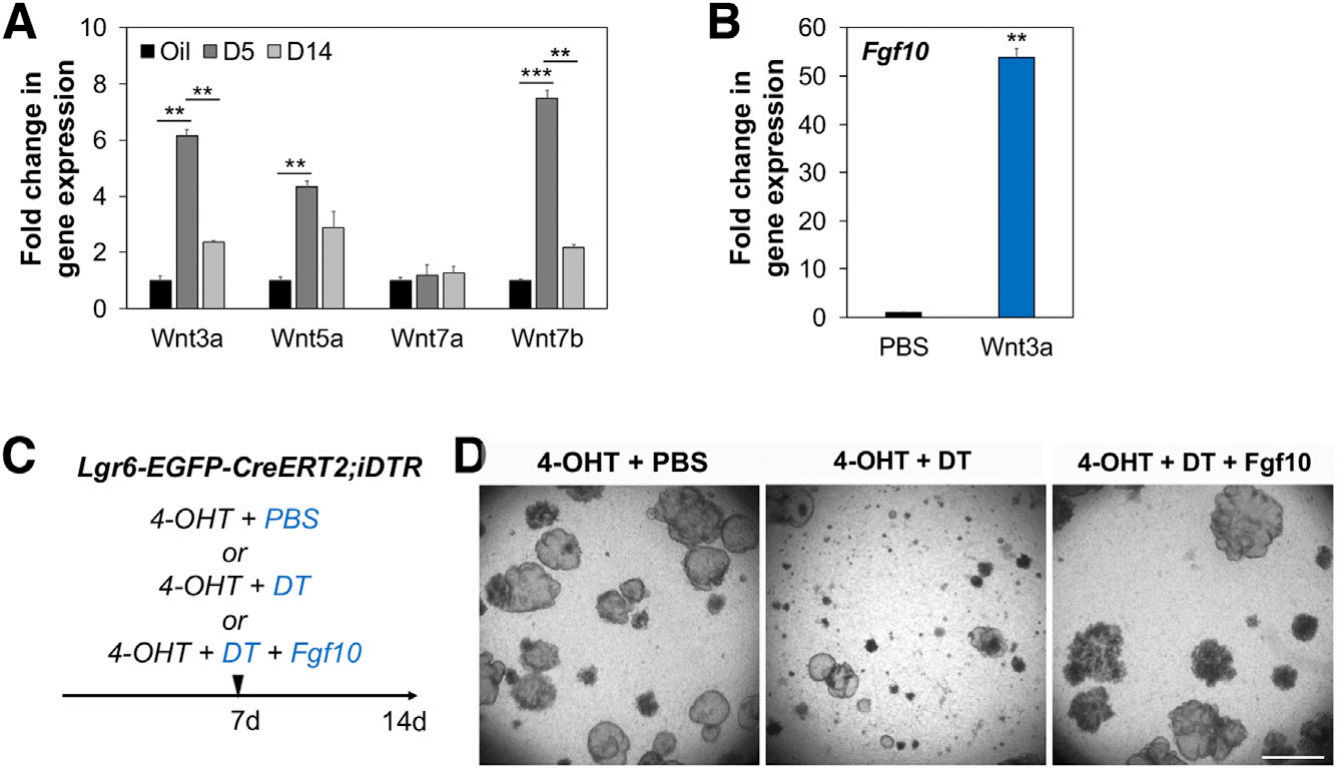
Lgr6^+^ Cells Produce Fgf10, which Acts on Proliferation and Differentiation of Scgb1a1^+^ Cells in In Vitro Organoid Formation, Related to Figure 6 (A) qPCR analysis for the expression of Wnt ligands in freshly isolated Scgb1a1^+^ cells from lung tissues of vehicle-treated and naphthalene-treated mice. Data presented are the mean of experiments from three individual mice per group. Error bars indicate standard deviation (^∗∗^p < 0.005; ^∗∗∗^p < 0.001). (B) qPCR analysis for the expression of *Fgf10* in cultured Lgr6^+^ cells with addition of PBS (black bar) and Wnt3a (blue bar). Normalized to *Gapdh*. Data presented are the mean of three independent experiments with triplicates. Error bars indicate standard deviation (^∗∗^p < 0.005). (C) Experimental scheme of depleting Lgr6-expressing cells in co-culture of Scgb1a1^+^ cells. *Lgr6-CreERT2;R26-iDTR* mice are utilized to isolate Lgr6-expressing cells following by co-culture with Scgb1a1^+^ cells. 4-OHT is added for inducing expression of DTR in Lgr6^+^ cells and DT is treated for ablation of these cells at day 7 in cultures with or without Fgf10. (D) Representative phase-contrast images of lung organoids with addition of DT and Fgf10 at day 7 in cultures. Scale bar, 500um.

Our in vitro data suggested the functional contribution of Lgr6^+^ cells to self-renewal and differentiation of epithelia (Figure 5). To determine whether this crosstalk between epithelial cells and Lgr6^+^ cells impacts airway regeneration in vivo, we investigated airway injury repair in the context of Lgr6^+^ cell depletion using the *Lgr6-EGFP-CreERT2*;*R26-iDTR*;*R26-Tom* mouse line. Naphthalene causes severe club cell depletion that is repaired within 7–30 days after injury. After final treatment of either DT or vehicle (PBS) to deplete lineage-labeled Lgr6^+^ cells, naphthalene was administered to ablate club cells (Figure 6A). IF analysis confirmed extensive club cell damage in PBS- and DT-treated mice 2 days after naphthalene administration (Figures 6B and 6C). We also observed efficient cell ablation of lineage-labeled Lgr6^+^ cells in the lungs delivered DT (Figure 6B). Five days after naphthalene administration, patches of club cells containing proliferating cells were observed in PBS control mice, yet club cell numbers in DT-treated mice remained low even at 10 days after club cell damage (Figures 6B–6D). Consistently, the number of club cells expressing Ki67 peaked at 5 days in response to naphthalene in control mice, whereas there was no discernible change in DT-treated mice (Figures 6B and 6D). Twenty days after club cell damage, there was very little remaining bronchiolar damage in mice administered PBS; however, significantly decreased numbers of club cells were still observed in mice administered DT (Figures 6B and 6C). Interestingly, there was a significant increase in Ki67 expression in lineage-labeled Lgr6^+^ cells surrounding the airways at 5 days post-naphthalene injury, when club cells are highly proliferative, in PBS control mice, yet DT-treated mice had fewer Ki67-positive lineage-labeled cells (Figures 6B and 6E), suggesting crosstalk between club cells and Lgr6^+^ cells during airway injury repair. Notably, upregulation of Wnt ligands was detected at 5 days post-naphthalene treatment in club cells and at 14 days post-injury returned to levels comparable to those in controls, suggesting that proliferating club cells release Wnt ligands that impact proliferation of Lgr6^+^ cells (Figure S5A).

Next, we asked whether Lgr6^+^ cells are required for maintaining capacity of club cells to grow and differentiate in culture. Lgr6^+^ cells were isolated from lungs in *Lgr6-EGFP-CreERT2*;*R26-iDTR* mice against GFP by FACS (CD31^−^CD45^−^EpCAM^−^GFP^+^) and co-cultured with lineage-labeled Scgb1a1^+^ cells isolated from *Scgb1a1-CreER*; *R26-YFP* animals. At day 1 in culture, 4-hydroxytamoxifen (OHT) was administered to Scgb1a1^+^/Lgr6^+^ co-cultures to induce DTR expression followed by adding either of DT or vehicle (PBS; Figure 6F). As expected, Scgb1a1^+^ cells grown in the presence of DT failed to form colonies, whereas those with PBS generated various types of colonies (Figures 6G and 6H). DT administration in Scgb1a1^+^/Lgr6^+^ co-cultures following vehicle (ethanol) treatment had no discernible effect, providing evidence for specific Lgr6^+^ cell depletion in our culture assay. Moreover, depletion of Lgr6^+^ cells at day 7 when each colony started developing distinct structures and inducing lineage differentiation resulted in impaired colony formation, including decreased numbers of colonies.

We next sought to identify key factors that are produced by Lgr6^+^ cells and support proliferation and maintenance of club cells. Given the contribution of Fgf10 to ASM progenitor cells during lung development (Volckaert and De Langhe, 2015), the expression level of *Fgf10* was assessed in Lgr6^+^ ASMCs. *Fgf10* was significantly upregulated at 5 days post-naphthalene injury and returned to control levels at 14 days post-injury (Figure 6I). We also confirmed that the level of *Fgf10* expression was greatly increased in proliferating Lgr6^+^ cells in response to Wnt activation (Figure S5B). In order to determine whether Fgf10 acts on club cell regulation, Fgf10 was added to Scgb1a1^+^/Lgr6^+^ co-cultures at day 7 when DT was treated to ablate Lgr6^+^ cells in cultures (Figure S5C). As expected, club cells started growing organoids yet failed to further proliferate and differentiate into airway and alveolar lineages when we depleted Lgr6^+^ cells at day 7 (Figure S5D). In contrast, addition of Fgf10 stimulated proliferation of club cells and partially rescued impaired organoid formation that was largely composed of bronchiolar and bronchioalveolar colonies (Figures 6J, 6K, and S5D). Together, these data provide strong evidence that Lgr6^+^ mesenchymal populations proliferate in response to airway damage and are required for proper airway injury repair partially via Fgf10-mediated regulation. Moreover, airway epithelial cells are important for inducing proliferation of Lgr6^+^ cells upon mesenchymal cell depletion by producing Wnt ligands, demonstrating that epithelial and mesenchymal cell crosstalk influences both cell types.

### Region-Specific Mesenchymal Regulation of Differentiation

**Figure 7:**
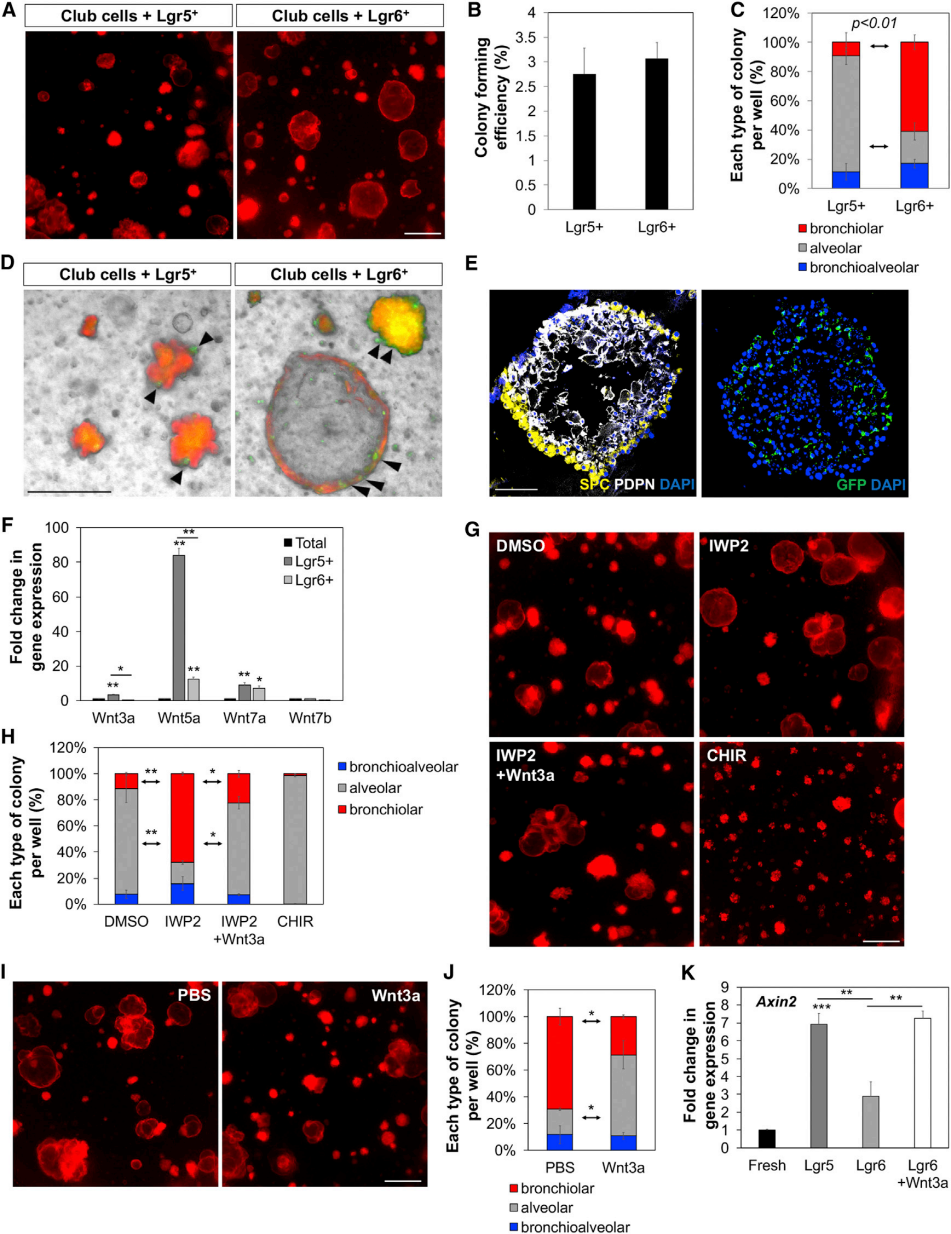
Distinct Role of Lgr5 and Lgr6 in Regulating Lineage Differentiation of Lineage-Labeled Scgb1a1^+^ Cells (A–D) Representative merged images of fluorescence (A) and phase contrast (D), colony forming efficiency (B), and quantification of each distinct type of primary lung organoids (C) from Scgb1a1^+^ cells co-cultured with Lgr5^+^ or Lgr6^+^ cells at 14 days in co-cultures. Arrowhead indicates close interactions of Lgr5^+^ or Lgr6^+^ cells (GFP, green) to club cell organoids (TdTomato, red). (E) Representative IF images of alveolar organoids from Scgb1a1^+^/Lgr5^+^ co-cultures. (Left) SPC (yellow), PDPN (white), and DAPI (blue) are shown. (Right) GFP (for Lgr5, green) and DAPI (blue) are shown. (F) qPCR analysis for expression of Wnt ligands in isolated total lung (black bar), Lgr5^+^ (dark gray bar), and Lgr6^+^ (light gray bar) cells. Shown is normalized to *Gapdh.* (G) Representative fluorescent images of Scgb1a1^+^/Lgr5^+^ co-cultures with addition of DMSO, IWP2, IWP2 and Wnt3a, and CHIR. (H) Quantification of each distinct type of colony from (G). (I) Representative fluorescent images of Scgb1a1^+^/Lgr6^+^ co-cultures with addition of PBS and Wnt3a. (J) Quantification of each distinct type of colony from (I). (K) qPCR analysis for expression of *Axin2* in freshly isolated Scgb1a1^+^ cells (black bar) and in Scgb1a1^+^ organoids co-cultured with Lgr5^+^ (dark gray bar), Lgr6^+^ (light gray bar), and Lgr6^+^ cells with Wnt3a treatment (white bar). Shown is normalized to *Gapdh*. Data presented are the mean of three independent experiments with triplicates (B, C, H, J, and K) or with three individual mice (F). Error bars indicate SD (^∗^p < 0.01; ^∗∗^p < 0.005; ^∗∗∗^p < 0.001). The scale bars represent 500 μm (A, G, and I) and 100 μm (D and E). See also Figure S6.

To address the specificity of the Lgr6^+^ cell effects on growth of epithelial cell organoids, we seeded Scgb1a1 lineage-labeled cells isolated from *Scgb1a1-CreER*; *R26-Tom* with Lgr6^+^ and Lgr5^+^ lung mesenchymal cells isolated from *Lgr6-EGFP-CreERT2* and *Lgr5-CreERT2*;*R26-EYFP* mice. Lgr5^+^ cells were sufficient for supporting club cell growth and differentiation; in Scgb1a1^+^/Lgr5^+^ co-cultures, CFE was similar to the co-culture of Scgb1a1^+^/Lgr6^+^ cells (Figures 7A and 7B). However, Lgr6^+^ cells enhanced bronchiolar colony formation and reduced alveolar colony formation in co-cultures compared to lineage-labeled Lgr5^+^ cells (Figures 5A and 5C). Of note, close interactions of Lgr5^+^ or Lgr6^+^ cells with epithelial organoids were observed (Figures 5D and 5E). IF revealed the presence of SPC-expressing AT2 cells and PDPN-expressing AT1 cells in alveolar organoids derived from club cells co-cultured with Lgr5^+^ cells, suggesting that Lgr5^+^ cells induce alveolar lineage differentiation of club cells (Figure 5E).

**Figure S6:**
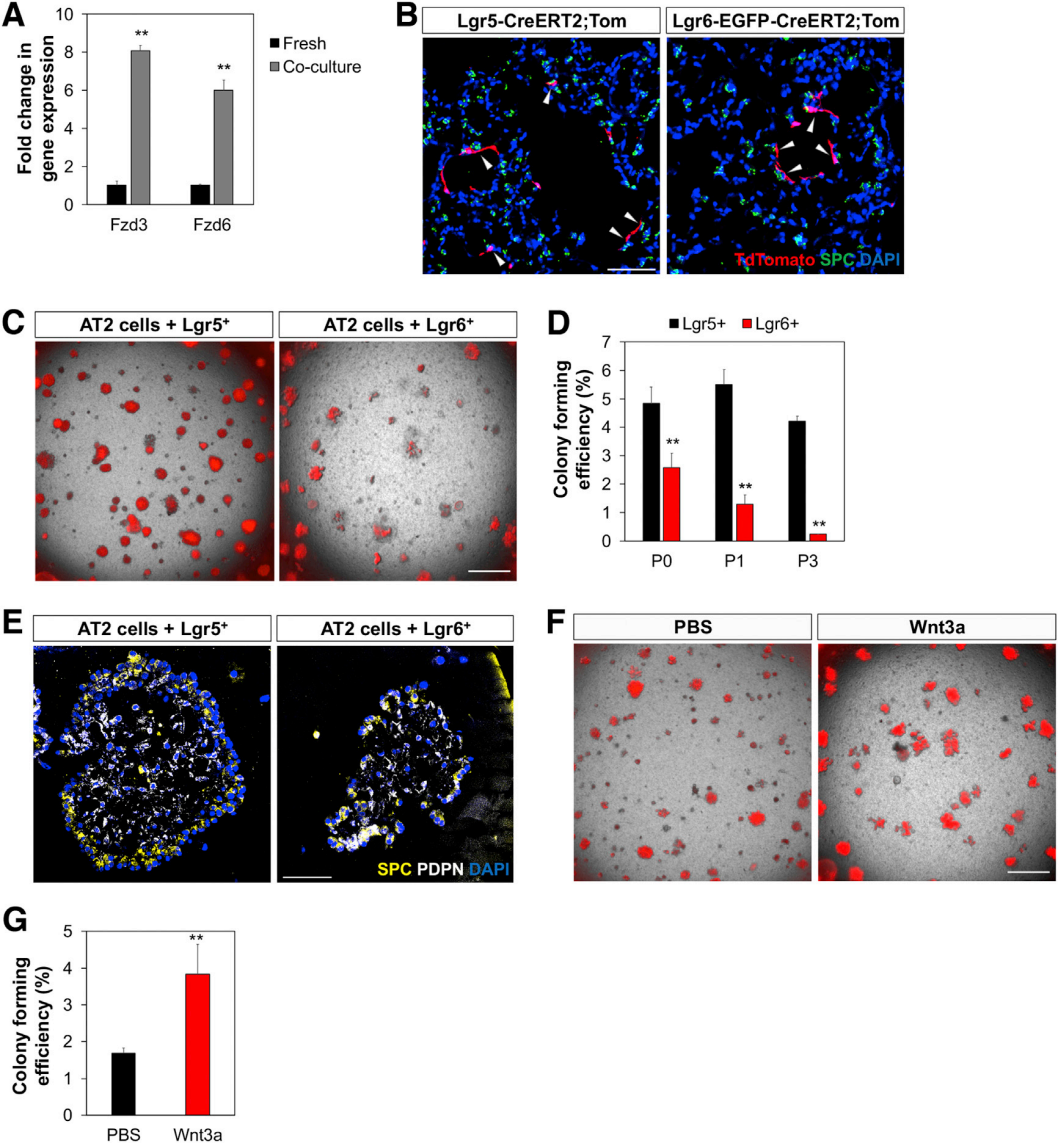
Contribution of Lgr5^+^ Cells to AT2 Cell Expansion and Differentiation, Related to Figure 7 (A) qPCR analysis for the expression of Fzd receptors in freshly isolated Scgb1a1^+^ cells from lung tissues and separated Scgb1a1^+^ cells from organoids co-cultured with Lgr5^+^ cells. Data presented are the mean of two independent experiments with triplicate wells. Error bars indicate standard deviation (^∗∗^p < 0.005). (B) Representative confocal images showing close interactions of lineage labeled Lgr5^+^ (left) and Lgr6^+^ (right) cells with AT2 cells in alveolar compartments: SPC (green), TdTomato (red), and DAPI (blue). Arrowheads indicate lineage labeled Lgr5^+^ and Lgr6^+^ cells located closely to AT2 cells. (C and D) Representative bright field-merged images (C) and colony forming efficiency (D) from Sftpc^+^ cells co-cultured with Lgr5^+^ (left) or Lgr6^+^ cells (right) at 14 days in co-cultures. Self-renewal ability of Sftpc^+^ cells was assessed; primary colonies (passage 0, P0) were dissociated and FACS sorted for EpCAM^+^Tom^+^ followed by replating with fresh Lgr5^+^/Matrigel or Lgr6^+^/Matrigel mixture for subsequent colony formation bi-weekly (passage 1, P1; passage 2, P2). Data presented are the mean of three independent experiments with triplicate wells. Error bars indicate standard deviation (^∗∗^p < 0.005). (E) Representative IF images of alveolar organoids from Sftpc^+^/Lgr5^+^ (left) and Sftpc^+^/Lgr6^+^ (right) co-cultures; SPC (yellow), PDPN (white), and DAPI (blue). (F and G) Representative brightfield-merged images (F) and colony forming efficiency (G) of Sftpc^+^/Lgr6^+^ co-cultures with addition of PBS (left) and Wnt3a (right). Data presented are the mean of three independent experiments with triplicate wells. Error bars indicate standard deviation (^∗∗^p < 0.005). Scale bar, 500um.

We next sought to identify key factors, produced by Lgr5^+^ cells, in regulating alveolar differentiation of club cells in this context. Wnt activity has been suggested to promote AT2 cell expansion during alveologenesis (Frank et al., 2016). However, studies have not yet examined a possible role for Wnt signaling in lineage-specific differentiation of adult lung progenitors. qPCR analysis for Wnt ligands revealed a marked increase in the expression of Wnt ligands, in particular *Wnt3a* and *Wnt5a*, in Lgr5^+^ cells compared to the expression levels in total lung cells and Lgr6^+^ cells (Figure 7F). We assessed whether activating Wnt signaling by secreted Wnt ligands would promote alveolar lineage differentiation. Treatment of Scgb1a1^+^/Lgr5^+^ organoids with the Wnt ligand secretion inhibitor IWP2 resulted in a significant increase in bronchiolar organoids, which was partially rescued with the addition of Wnt3a to the culture media (Figures 7G and 7H). Addition of a Wnt agonist CHIR stimulated alveolar differentiation of club cells. These data indicate that secreted Wnt ligands from Lgr5^+^ cells induce alveolar lineage differentiation of epithelial progenitors. Notably, treatment of Scgb1a1^+^/Lgr6^+^ organoids with Wnt3a resulted in a significant increase in alveolar organoid formation compared to co-cultures with PBS control (Figures 7I and 7J). We also confirmed that the level of *Axin2* is higher in organoids co-cultured with Lgr5^+^ cells compared to organoids co-cultured with Lgr6^+^ cells (Figure 7K). Addition of Wnt3a to club/Lgr6 organoids resulted in an increase in *Axin2* expression levels, indicating Wnt pathway activation in these cells. To identify the Frizzled (Fzd) receptors that may be involved in Wnt-mediated alveolar differentiation, we performed qPCR on Fzd receptors in freshly sorted club cells and club cells that were co-cultured with Lgr5^+^ cells for 7 days. *Fzd3* and *Fzd6* were robustly expressed in freshly isolated club cells and co-culture with Lgr5^+^ cells resulted in a marked increase of their expression in club cells (Figure S6A). These data strongly suggest that Wnt activity induces alveolar lineage differentiation.

Given the close proximity of Lgr5^+^ and Lgr6^+^ cells to AT2 cells in alveolar compartments (Figure S6B), we also examined whether Lgr5^+^ or Lgr6^+^ cells support AT2 cell growth in organoid co-culture system. Isolated lineage-labeled AT2 cells from *Sftpc-CreERT2*;*R26-Tom* mice were co-cultured either with Lgr5^+^ or Lgr6^+^ cells (Figure S6C). AT2 cells co-cultured with Lgr5^+^ cells showed greater organoid formation compared to AT2 cells co-cultured with Lgr6^+^ cells (Figure S6D). Lgr5^+^ cells support the self-renewal ability of AT2 cells without decreased CFE with multiple passages, whereas the CFE of AT2 cells with Lgr6^+^ cells decreased with passage. Both Lgr5^+^ and Lgr6^+^ cells also supported multi-lineage differentiation of AT2 cells, as noted by expression of SPC and PDPN for AT2 and AT1 cells, respectively, whereas organoids were slightly bigger when AT2 cells co-culture with Lgr5^+^ cells than with Lgr6^+^ cells (Figure S6E). There was no airway differentiation of lineage-labeled AT2 cells even in co-culture with Lgr6^+^ cells. We next assessed whether activating Wnt signaling would also result in enhanced organoid formation of AT2 cells. Adding Wnt3a to Sftpc^+^/Lgr6^+^ organoids showed a marked increase in organoid formation, suggesting that Wnt signaling is also important for AT2 cell expansion (Figures S6F and S6G). Together, these data identify mesenchymal Lgr6^+^ cells as a stromal cell subtype that preferentially supports airway differentiation, whereas Lgr5^+^ cells promote alveolar differentiation. Given their close proximity to epithelial cells in vivo, these results strongly support the idea that region-specific mesenchymal cell subsets are a critical input for driving lineage maintenance and specification of epithelial progenitors.

## Discussion

The importance of mesenchymal cells in development, homeostasis, and injury repair is well appreciated in the lung and many other tissues types, yet the identity of specific mesenchymal cell subsets and how they influence epithelial cells remain unknown. We show that adult lung mesenchymal cells can be distinguished phenotypically and functionally on the basis of Lgr5 and Lgr6. Proliferation and repair of different lung epithelial cell types is reliant on specific mesenchymal cell partners. In turn, mesenchymal cell dynamics are influenced by epithelial cells, demonstrating the co-dependencies between these cell types in the adult lung.

Active crosstalk between epithelial and mesenchymal cells has been demonstrated during lung development, supporting our findings in adult lung. Mesenchymal progenitors emerge, migrate, and govern the formation of distinct cell types in the lung niches. Fgf10 expression in the distal mesenchyme acts on adjacent distal buds (Abler et al., 2009, Bellusci et al., 1997). Wnt signaling in distal mesenchyme is also a well-established driver of lung development (Cardoso and Lü, 2006, Morrisey and Hogan, 2010). Shh-Ptch signaling in epithelial and mesenchymal cells contributes to multiple lineages of mesenchymal cells and lung morphogenesis (Cardoso and Lü, 2006, Li et al., 2015, Peng et al., 2015).

Our studies indicate that epithelial and mesenchymal cell types form specific partnerships in the adult lung. Our findings expand on strong previous evidence for paracrine signaling between ASMCs and epithelia (Volckaert et al., 2011, Volckaert et al., 2013). Lgr6^+^ cells contribute to quiescence, which is also governed by Gli1-expressing cells. The Lgr5^+^ mesenchymal cells, which we found support alveolar differentiation, appear to be distinct from Pdgfrα^+^ cells (Figures 2C–2E), which have been known to promote AT2 self-renewal and differentiation (Barkauskas et al., 2013). Complimentary studies reported by Zepp et al. (2017) show that Wnt-responsive mesenchymal cell populations expressing Pdgfrα (Axin2^+^Pdgfrα^+^ mesenchymal alveolar niche cells [MANCs]) are found close to AT2 cells and enhance their expansion whereas Axin2^+^Pdgfrα^−^ cells contribute to fibrogenic myofibroblasts. MANCs and the Axin2^+^ myofibrogenic progenitors may be the two different Lgr5-expressing alveolar mesenchymal cell populations we found in two clusters with different transcriptional programs; further studies will be required to determine whether these clusters represent cells types with distinct functional properties. Lgr6-expressing ASMCs may be derived from an Axin2^+^ progenitor near the airway described by Zepp et al. Understanding the complex interplay of these different cell types will require further analysis. Finally, although previous studies reported E-cadherin^+^ Lgr6^+^ cells as an epithelial progenitor population in human lung (Oeztuerk-Winder et al., 2012), we find that the majority of Lgr6-expressing cells in the adult murine lung are mesenchymal. However, the murine equivalent of the human Lgr6^+^ progenitor population may be reflected by a distinct small population expressing EpCAM revealed by our scRNA-seq data and lineage-tracing studies (Figures 2B–2E and S2G–S2I).

Our lineage-tracing studies indicate that subsets of ASMCs labeled by Lgr6 are capable of low proliferation rate and ASMCs are dynamic after cellular damage. In lung development, a subset of Fgf10-expressing cells in the distal mesenchyme are progenitor cells for ASMCs. Tight regulation of Fgf10 and Wnt ligand expression during lung development is critical for controlling a balance between proliferation and lineage differentiation (Volckaert and De Langhe, 2015). In the adult lung, our studies suggest that a paracrine Wnt–Fgf10 signaling cascade between club cells and Lgr6^+^ cells occurs in the airway injury repair process (Figure S5A).

We characterized some of the distinct features of Lgr5^+^ and Lgr6^+^ cells that act on lineage differentiation of epithelial progenitor cells in adult lungs by analysis of Wnt activity. Recent studies showed that a Wnt-responsive subpopulation of AT2 cells has a greater clonal output than the bulk AT2 population during post-natal growth (Frank et al., 2016). Our data from genetic models and organoid cultures indicate that Lgr5^+^ cells induce alveolar lineage differentiation and Lgr6^+^ cells preferentially support bronchiolar lineages, consistent with the anatomic location of the Lgr5^+^ or Lgr6^+^ cells. Notably, pharmacological perturbation of Wnt production suppressed alveolar organoid formation, indicating that Wnt ligands derived from Lgr5^+^ cells drive alveolar lineage differentiation. Given our observation of Wnt ligand production in proliferating club cells, a positive feedback Wnt signal from epithelial cells may also regulate differentiation. Future studies will address how Wnt signals operate between mesenchymal and epithelial cell types to maintain epithelial integrity and differentiation specificity in the airway and the alveolar space.

Our findings may lead to new specific opportunities for therapeutic interventions across diverse lung diseases. Targeting the Lgr5^+^ mesenchymal cells in lung may influence alveolar disease, whereas Lgr6^+^ mesenchymal cells may be a specific target for airway disease. Smooth muscle cells have been known to regulate airway epithelial cell behavior; they are the major target of some types of therapy for asthma. Our identification of the specific Lgr5/6 mesenchymal cellular partnerships with epithelial cells provides new ways to understand the complexity of how different cell types are maintained in the healthy lung and possible mechanisms that likely go awry in lung disease.

## STAR★Methods

### Key Resources Table

**Table:** 

REAGENT or RESOURCE	SOURCE	IDENTIFIER
**Antibodies**		

CD45-APC	BD Biosciences	Cat# 559864; RRID: AB_398672
CD31-APC	BD Biosciences	Cat# 551262; RRID: AB_398497
Ter119-APC	BD Biosciences	Cat# 557909; RRID: AB_398635
CD11b-APC	BD Biosciences	Cat# 553312; RRID: AB_398535
EpCAM-PE-Cy7	BioLegend	Cat# 118216; RRID: AB_1236471
DAPI (4′, 6-diamidino-2-phenylindole)	Sigma	Cat# D9542
GFP (chick)	Abcam	Cat# ab13970; RRID: AB_300798
SFTPC (goat)	Santa Cruz Biotechnology	Cat# sc-7706; RRID: AB_2185507
CC10 (rabbit)	Santa Cruz Biotechnology	Cat# sc-25555; RRID: AB_2269914
CC10 (goat)	Santa Cruz Biotechnology	Cat# sc-9772; RRID: AB_2238819
Ki67 (rabbit)	Abcam	Cat# ab16667; RRID: AB_302459
Ki67 (mouse)	BD Biosciences	Cat# 550609; RRID: AB_393778
RFP (rabbit)	Rockland	Cat# 600-401-379; RRID: AB_2209751
α-Smooth Muscle Actin (mouse)	Sigma	Cat# A5228; RRID: AB_262054
PDPN (hamster)	DSHB	Cat# 8.1.1; RRID: AB_531893

**Chemicals, Peptides, and Recombinant Proteins**		

Tamoxifen	Sigma	Cat# T5648
Mazola corn oil	Sigma	Cat# C8267
Diphtheria toxin	Sigma	Cat# D0564
Growth factor reduced matrigel	BD Biosciences	Cat# 354234
Dimethyl Sulfoxide	Sigma	Cat# D2650
IWP2	Stemgent	Cat# 130-105-335
CHIR99021	Tocris	Cat# 4423
ITS	Corning	Cat# 25-800-CR
Y27632	Sigma	Cat# Y0503
hFgf10	Peprotech	Cat# 100-26
4-hydroxytamoxifen	Sigma	Cat# H7904

**Deposited Data**		

Single Cell RNA Sequencing	This paper	GEO: GSE101334

**Experimental Models: Cell Lines**		

Mouse L cells	PMID: 12717451	N/A

**Experimental Models: Organisms/Strains**		

Rosa26-lox-stop-lox-YFP	Jackson Laboratories	Cat# 006148
Rosa26-lox-stop-lox-TdTomato	Jackson Laboratories	Cat# 007914
Rosa26-lox-stop-lox-DTR	Jackson Laboratories	Cat# 007900
Scgb1a1-CreERTM	PMCID: PMC2730729	N/A
Sftpc-CreERT2	Jackson Laboratories	Cat# 028054
Lgr6-EGFP-IRES-CreERT2	Jackson Laboratories	Cat# 016934
Lgr5-IRES-CreERT2	PMCID: PMC3634804	N/A

**Oligonucleotides**		

Gapdh (Mm00805216_m1)	Life Technologies	Cat# 4331182
Lgr5 (Mm00438890_m1)	Life Technologies	Cat# 4331182
Lgr6 (Mm01291336_m1)	Life Technologies	Cat# 4331182
Acta2 (Mm00725412_s1)	Life Technologies	Cat# 4331182
Scgb1a1 (Mm00442046_m1)	Life Technologies	Cat# 4331182
Foxj1 (Mm01267279_m1)	Life Technologies	Cat# 4331182
Muc5ac (Mm01276735_m1)	Life Technologies	Cat# 4331182
Sftpc (Mm00488144_m1)	Life Technologies	Cat# 4331182
Abca3 (Mm00550501_m1)	Life Technologies	Cat# 4331182
Lamp3 (Mm00616604_m1)	Life Technologies	Cat# 4331182
Ager (Mm01134790_g1)	Life Technologies	Cat# 4331182
Hopx (Mm00558629_m1)	Life Technologies	Cat# 4331182
Ki67 (Mm01278617_m1)	Life Technologies	Cat# 4331182
Primers for Sybr green assays in this study, see STAR Methods	This paper	N/A

**Software and Algorithms**		

GraphPad Prism	GraphPad Software, Inc	http://www.graphpad.com/scientific-software/prism/
Fiji	N/A	https://imagej.net/Fiji
FlowJo	FlowJo, LLC	https://www.flowjo.com/solutions/flowjo
MATLAB R2016b	Mathworks Ltd	N/A
R V3.3	CRAN	N/A

### Contact for Reagent and Resource Sharing

Further information and requests for resources and reagents should be directed to and will be fulfilled by the Lead Contact, Carla Kim (Carla.kim@childrens.harvard.edu).

### Experimental Model and Subject Details

#### Mouse Strains

All mice work was approved by the CHB Animal Care and Use Committee, accredited by AAALAC, and was performed in accordance with relevant institutional and national guidelines and regulations. *Lgr6-EGFP-IRES-CreERT2*, *Sftpc-CreERT2*, *Rosa26-lox-stop-lox-tdTomato*, *Rosa26-lox-stop-lox-YFP,* and *Rosa26-lox-stop-lox-DTR* mice were purchased from Jackson Laboratories. The *Scgb1a1-CreER*^*TM*^ (Rawlins et al., 2009) and *Lgr5-IRES-CreERT2* (Huch et al., 2013) mouse lines were kindly provided by Dr. Brigid Hogan and by Dr. Hans Clevers, respectively. Mice for the lineage tracing and injury experiments were on a C57BL/6 background and controls matched for sex and were littermates. 7-10 week old mice were used for most of experiments described in this study. Animal studies were reviewed and approved by the Massachusetts Institute of Technology (MIT) Committee for Animal Care (institutional animal welfare assurance no. A-3125-01) or the Boston Children’s Hospital’s Institutional Animal Care and Use Committee.

#### Lung organoid co-cultures

7-10 week old mice were used to generate lung organoids, previously reported (Lee et al., 2014). Briefly, freshly sorted lineage-labeled Scgb1a1^+^ or Sftpc^+^ cells were resuspended in 3D basic media, and mixed with Lgr5^+^ or Lgr6^+^ cells containing growth factor-reduced Matrigel (BD Biosciences) at a ratio of 1:1; 100 μL of mixtures was placed in a 24-well Transwell insert with a 0.4-μm pore (Corning). In some experiments, sorted Lgr5^+^ and Lgr6^+^ cells were seeded in a collagen-coated plate (Corning) and expanded for 5-7 days for further organoid co-culture assays with Scgb1a1^+^ or Sftpc^+^ cells. Approximately 0.5-1 × 10^4^ Scgb1a1^+^ or Sftpc^+^ cells and 0.5-1 × 10^5^ Lgr5^+^ or Lgr6^+^ cells were seeded in each insert. 500 μL of 3D basic media was placed in the lower chamber, and medium was changed every other day with or without 4-hydroxytamoxifen (500nM, Sigma), DT (50ng/mouse, Sigma), DMSO (Sigma), IWP2 (1.5 μM, Stemgent), CHIR99021 (3 μM, Tocris), rhFgf10 (10ng/ml, Peprotech), rmWnt3a (100ng/ml, R&D) and Wnt3a conditioned media (50%, produced using stably transfected L cells). 3D basic medium: Dulbecco’s Modified Eagle’s Medium/F12 (Invitrogen) was supplemented with 10% FBS, penicillin/streptomycin, 1 mM HEPES, and insulin/transferrin/selenium (ITS) (Sigma). ROCK inhibitor Y27632 (10uM, Sigma) was included in the medium for the first 2 days of culture, which was at 37°C in 7% CO_2_/air. For serial passages, organoids were dissociated in dispase (BD Bioscience) and trypsin (GIBCO) to generate a single-cell suspension followed by FACS for EpCAM^+^. EpCAM^+^ cells were resuspended with fresh Lgr/Matrigel mixtures for subsequent colony formation bi-weekly. Plates were scored for numbers of colony after 14 days. Colony forming efficiency was calculated the number of colonies formed/number of cells plated per well as a percentage.

### Method Details

#### Tamoxifen, Diphtheria Toxin and Naphthalene Administration

Tamoxifen (Sigma) was a 20 mg/ml stock solution in Mazola corn oil (Sigma) and given via intraperitoneal (IP) injection. 7-10 week old *Lgr6-EGFP-CreERT2;R26-Tom;R26-iDTR* mice were injected intratracheal with DT (Sigma) at a dose of 50ng/mouse dissolved in PBS. *Scgb1a1-CreERT2;R26-YFP* mice or DT/PBS-treated *Lgr6-EGFP-CreERT2;R26-Tom;R26-iDTR* mice were administered with a naphthalene (Sigma) at a dose of 250mg/kg dissolved in Mazola corn oil via IP injection. At indicated time points, lungs were collected for isolating lung cells or histological analysis.

#### Mouse Lung Dissociation and Flow Cytometry

Lungs were dissociated with a collagenase/dispase solution as previously described (Lee et al., 2014). Briefly, after lungs were cleared of blood by perfusion with cold PBS through the right ventricle, 2 mL of dispase (BD Biosciences, 50 U/ml) were instilled into the lungs through the trachea until the lungs inflate, and follow with instillation of 1% low melting agarose (BioRad) through the trachea to prevent leakage of dispase. Each of lobes were dissected off and minced into small pieces in a conical tube containing 3ml of PBS, 60 μL of collagenase/dispase (Roche), and 7.5 μL of 1% DNase I (Sigma) followed by rotating incubation for 45 min at 37°C. The cells were then filtered sequentially through 100- and 40-μm strainers and centrifuged at 1000rpm for 5 min at 4°C. The cell pellet was resuspended in 1ml of RBC lysis buffer (0.15 M NH4Cl, 10mM KHCO3, 0.1 mM EDTA) and lysed for 90 s at room temperature. Addition of 6ml basic F12 media (GIBCO) was followed and 500 μL of FBS (Hyclone) was slowly added in the bottom of tube. Cells were centrifuged at 1000rpm for 5 min at 4°C. The cell pellet was resuspended in PBS with 10% FBS for further staining against antibodies for mouse flow cytometry: pan CD45-APC, CD31-APC, CD11b-APC, Ter119-APC (BD Biosciences), and EpCAM-PE-Cy7 (BioLegend). 4’, 6-diamidino-2-phenylindole (DAPI) (Sigma) was used to eliminate dead cells. Cell sorting was performed with a FACS Aria II (BD Biosciences) and a Moflo Astrios Eq (Beckman Coulter), and data were analyzed with FlowJo software (Tree Star, Inc.).

#### Histology and Immunohistochemistry

Mouse lung tissues were routinely perfused, inflated, and fixed with 4% paraformaldehyde (PFA) for 4 hr at room temperature and cryosections (12um) and paraffin sections (6um) were used for histology and immunofluorescent (IF) analysis. Cultured colonies were fixed with 4% PFA for 2-4 hr at room temperature followed by immobilized with Histogel (Thermo Scientific) for paraffin embedding. Sectioned lung tissues or colonies were stained with hematoxylin and eosin (H&E) or immunostained: after antigen retrieval with citric acid (0.01M, pH = 6), blocking was performed with 5% normal donkey serum in 0.2% Triton-X/PBS at room temperature for 60 min. Primary antibodies were incubated overnight at 4°C at the indicated dilutions: chicken anti-GFP (1:500, Abcam, ab13970), goat anti-SP-C (1:200, Santa Cruz Biotechnology Inc., sc-7706), rabbit anti-CC10 (1:200, Santa Cruz Biotechnology Inc., sc-25555), rabbit anti-Ki67 (1:500, Abcam, ab15580), mouse anti-Ki67 (1:500, BD Biosciences, 550609), rabbit anti-RFP (for tdTomato) (1:250, Rockland, 600–401379), mouse anti-α Smooth Muscle Actin (1:1000, Sigma, A2547), hamster anti-PDPN (1:1000, DSHB, 8.1.1). Alexa Fluor-coupled secondary antibodies (1:500, Invitrogen) were incubated at room temperature for 60 min. After antibody staining, nuclei were stained with Hoechst dye (1:1000, ThermoFisher) and sections were embedded in Vectashield (Vector Labs). For whole-mount staining, PFA fixed lung tissues were embedded in 3% low melt agarose, followed by sectioned into 100-150um thick slices. After antibody incubation for 3-6 days in PBS with 0.2% Triton X-100, Scale A2 reagent was used for clearing the slices for 1 week at 4°C. Bright-field images were acquired using a EVOS microscope (ThermoFisher Scientific). Fluorescence images were acquired using a confocal microscope (Leica TCS SP5). All the images were further processed with Fiji software.

#### RNA extraction and quantitative RT–PCR

Total RNA from mouse lung tissues or sorted cells was prepared using Trizol reagent (Thermo Fisher Scientific). Briefly, the cell pellets were resuspended in 500 μL of Trizol and added 100 μL of chloroform followed by vortexing for 15 s. After 5 min incubation at room temperature, samples were centrifuged at 12,000rpm for 15 min at 4°C. Following centrifugation, the aqueous phase that retains RNA was transferred into fresh tube without disturbing the interphase. RNA precipitation from the aqueous phase was performed by adding and mixing with 250 μL of isopropyl alcohol. Samples were incubated for 10 min at room temperature and centrifuged at 12,000rpm for 10 min at 4°C. The RNA was pelleted and washed once in 80% ethanol. The air-dried pellet was resuspended in DEPC-treated water. Double-stranded cDNA was generated with Superscript III First-Strand Synthesis kit (Thermo Fisher Scientific) by manufacturer’s instructions. Real-time PCR amplification and analysis was conducted in StepOneTM Real-Time PCR Systems (Thermo Fisher Scientific) and performed in triplicate with a standard curve for every primer. Pre-designed probe sets and TaqMan Universal PCR Master Mix (2x) (Thermo Fisher Scientific) were used as follows: *Lgr5* (Mm00438890_m1), *Lgr6* (Mm01291336_m1), *Acta2* (Mm00725412_s1), *Scgb1a1* (Mm00442046_m1), *Foxj1* (Mm01267279_m1), *Muc5ac* (Mm01276735_m1), *Sftpc* (Mm00488144_m1), *Abca3* (Mm00550501_m1), *Lamp3* (Mm00616604_m1), *Ager* (Mm01134790_g1), *Hopx* (Mm00558629_m1), and *Ki67* (Mm01278617_m1). *Gapdh* expression (Mm00805216_m1) was used to normalize samples using the ΔCt method. Sybr green assays were also used with SYBR Green Master Mix (2x) (Thermo Fisher Scientific). *Gapdh* was used for normalization.

Primer sequences:

Gapdh F GGTGAAGGTCGGTGGAACG Gapdh R CTCGCTCCTGGAAGATGGTG
iDTR F GGAGCACGGGAAAAGAAAG iDTR R GAGCCCGGAGCTCCTTCACA
Axin2 F TGACTCTCCTTCCAGATCCCA Axin2 R TGCCCACACTAGGCTGACA
Wnt3a F ACCGTCACAACAATGAGGCT Wnt3a R TCGGCACCTTGAAGTACGTG
Wnt5a F CAACTGGCAGGACTTTCTCAA Wnt5a R CCTTCTCCAATGTACTGCATG
Wnt7a F GGCTTCTCTTCGGTGGTAGC Wnt7a R TGAAACTGACACTCGTCCAGG
Wnt7b F CTTCACCTATGCCATCACGG Wnt7b R TGGTTGTAGTAGCCTTGCTTCT
Fzd3 F ATGGCTGTGAGCTGGATTGTC Fzd3 R GGCACATCCTCAAGGTTATAGG
Fzd6 F TCTGCCCCTCGTAAGAGGAC Fzd6 R GGGAAGAACGTCATGTTGTAAGT
Fgf10 F TCAGTGGAAATCGGAGTTGT Fgf10 R TGCTGCCAGTTAAAAGATGC

#### Single Cell Sequencing

We used a modified version of the SmartSeq2(SS2) protocol (Picelli et al., 2013), as previously described (Shekhar et al., 2016). Single cells were sorted in 96 well plates, in lysis buffer (TCL 1%BME). We used Agencourt RNA-Clean strepdavadin beads (Beckman Coulter) to precipitate nucleic acids, which were cleaned by washing with 70% ethanol. RNA extraction step is done with Agilent Bravo Automated Liquid Handlin Platform. Next, we performed reverse transcription of polyadenylated transcripts using an oligo-dT primer and a reverse transcriptase derived from the Moloney murine leukemia virus (MMLVRT), followed by a a template switching reaction that relies on the terminal-transferase activity of the MMLVRT in the presence of a template switch oligonucleotide primer (TSO). The double-stranded RT-product was PCR amplified using Kapa Ready Mix (Kapa Biosystems) for 21 cycles, to yield the whole transcriptome amplification (WTA) product. The WTA product was cleaned up with AMPure SPRI beads and 80% ethanol, and QCed with BioAnalyzer (to confirm the correct product size) and qubit (to determine quantity). Next, we incubate the WTA product with Tn5 transposase, using the dual-index strategy from Illumina. Each single-cell library was individually barcoded by PCR with index primers. The barcoded single cells were pooled and sequenced on an Illumina NextSeq sequencer.

### Quantification and Statistical Analyses

#### Statistical Analysis

Statistical methods relevant to each figure are outlined in the figure legend. Statistical analyses were performed with Prism software package version 6.0 (GraphPad). *P* values were calculated using two-tailed unpaired or paired Student’s t test. Sample size for animal experiments was determined based upon pilot experiments. Mice cohort size was designed to be sufficient to enable accurate determination of statistical significance. No animals were excluded from the statistical analysis, unless due to technical errors. Mice were randomly assigned to treatment or control groups, while ensuring inclusion criteria based on gender and age. Investigators were blinded for all tissue staining and quantifications. Appropriate statistical analyses were applied, assuming a normal sample distribution. Data shown are either representative of three or more independent experiments or combined from three or more independent experiments as noted and analyzed as mean ± SEM.

#### Analysis of Single Cell Sequencing

Following sequencing, the 38bp paired-end reads were pseudo-aligned to the mm10 mouse transcriptome using Kallisto (Bray et al., 2016), with Kmers of length 31, and transcript counts were calculated and summed to gene counts. Cells were excluded from further analysis based on the following exclusion criteria: (1) The number of expressed genes falls below 2000, (2) they exhibit a very low mean expression in a panel of house-keeping genes (TPM < 1.5), (3) they show an outlier number of expressed genes (top 1%), (4) they show sufficiently high expression of *R26-Tom* (TPM > 32), (5) they show no expression of CD45 (Ptprc, TPM < 1), (6) they show no expression of CD31 (Pecam1, TPM < 1). After applying these criteria we are left with 182 cells for further analysis, of these 57 were Lgr5^+^ (CD31^–^CD45^–^CD11b^–^TER119^–^Tom^+^), and 125 were Lgr6^+^ (CD31^–^CD45^–^CD11b^–^TER119^–^GFP^+^). Next, the counts of individual cells were scaled to a sequencing depth of 100,000 reads per cell, using a scalar scaling factor calculated by sampling the reads from individual cells and fitting the original counts to the re-sampled counts with robust linear regression. Next, we use the Seurat R package (version 1.4.0.6) (Satija et al., 2015), to identify genes exhibiting elevated dispersion (> 0.5). We use consensus clustering (Wilkerson and Hayes, 2010), an unsupervised clustering technique for identifying robust clusters, based on 1000 runs of a community-detection clustering algorithm on a k-NN graph of the cells, as described previously (Shekhar et al., 2016). Using this approach, we identify 5 robustly occurring clusters by examination of the consensus clustering co-occurrence matrix (as in Figure 2D). Single cells transcriptional state and cluster subtypes were visualized post hoc using t-stochastic neighborhood embedding with which we generated a 2D embedding of the data based on the 7 leading principle components and perplexity of 20, as previously described (Macosko et al., 2015).

#### Cell Counting and Image Analysis

Sections included in cell scoring analysis were acquired using Leica TCS SP5 confocal microscope. At least five different sections including at least 25 bronchioles and 15 alveolar regions from three individual mice per group were used. Cell counts were performed on ImageJ using the ‘Cell Counter’ plug-in and the performer was blinded to the specimen genotype and condition. Quantification of distinct types of differentiated colonies was performed by scoring the colonies expressing CCSP or SPC by IF staining from at least five step sections (20um apart) per individual well.

### Data and Software Availability

The accession number for the single cell sequencing datasets reported in this paper is GEO: GSE101334.

## Author Contributions

J.-H.L. and C.F.K. designed the experiments and wrote the manuscript with input from co-authors. J.-H.L. performed all experiments and data analysis. T.T. provided lung tissues from *Lgr5* mice. T.T., N.D.M., D.C., and K.W. performed scRNA-seq experiments. M.H. and S.H. analyzed scRNA-seq data. J.C. performed organoid culture experiments. M.P. provided *Lgr6* mice for scRNA-seq experiments. D.H.B. shared gene expression data. J.-H.L., T.J., A.R., and C.F.K. secured funding. All authors discussed the results and commented on the manuscript.
